# How Much Variation Can One Ant Species Hold? Species Delimitation in the *Crematogaster kelleri*-Group in Madagascar

**DOI:** 10.1371/journal.pone.0068082

**Published:** 2013-07-09

**Authors:** Bonnie B. Blaimer, Brian L. Fisher

**Affiliations:** 1 Department of Entomology, Smithsonian Institution, Washington, D.C., United States of America; 2 Department of Entomology, California Academy of Sciences, San Francisco, California, United States of America; 3 Department of Entomology, University of California Davis, Davis, California, United States of America; Université Paris 13, France

## Abstract

We investigated the species-level taxonomy of the Malagasy *Crematogaster* (*Crematogaster*) *kelleri*-group and an additional more distantly related species of the same subgenus. Morphological data from worker, queen and male ants, as well as genetic data from three nuclear genes (long wavelength rhodopsin, arginine kinase and carbomoylphosphate synthase) and one mitochondrial marker (cytochrome oxidase I) led to the recognition of six species. Within the *C. kelleri*-group, three new species are described: *C. hazolava* Blaimer **sp. n.**, *C. hafahafa* Blaimer **sp. n.** and *C. tavaratra* Blaimer **sp. n.** The previously described taxa *C. kelleri* Forel and *C. madagascariensis* André are validated by our analysis. Conversely, our data suggests synonymy of *C. adrepens* Forel (with *C. kelleri*) and *C. gibba* Emery (with *C. madagascariensis*). A more distantly related and phylogenetically isolated species, *C. tsisitsilo* Blaimer **sp. n.**, is further described. We report high levels of morphological and molecular variation in *C. kelleri* and illustrate that this variation can be explained partly by geography. Species descriptions, images, distribution maps and identification keys based on worker ants, as well as on queen and male ants where available, are presented for all six species. Our work highlights the elevated species richness of *Crematogaster* ants throughout Madagascar’s humid forests, especially in the far northern tip of the island, and the need to use multiple data sources to ensure clear demarcation of this diversity.

## Introduction

The island of Madagascar is home to such a diverse array of endemic creatures that is sometimes called the “eighth continent”. However, when this extraordinary diversity is described, the most diverse group of organisms all too frequently is not even mentioned; the arthropods of Madagascar show species-richness and endemism levels unrivalled by any other terrestrial animal group on this island [1]. That said, extreme diversity can sometimes be a drawback. Many arthropod groups in Madagascar are still little known, and information on their systematics, distribution and natural history is fragmentary. Ants especially have been a focus of insect biodiversity research in Madagascar for many years [2]–[4], yet we still lack an accurate estimate of the number of ant species supported by the island. The large numbers of species to describe render the progress of taxonomic research particularly slow-moving. Accurate species delimitation and distribution records, however, form the basis for all subsequent research and conservation questions in Madagascar – the answers to which must be found quickly because natural habitats are shrinking [5].

Ant taxonomists are frequently faced with an additional challenge to their work: exceptional intraspecific morphological variation. Such morphological variety often complicates species delimitation and augments the amount of data and time needed to make well-supported decisions. Historical misinterpretation of this variation has often led to nomenclature problems that need to be resolved in the context of taxonomic revisions [6]. One group of ants infamous for its morphological variability is the genus *Crematogaster* Lund. Commonly known as acrobat ants, the group has almost 500 described species and about 300 subspecies names [7], [8]. Distributed worldwide, the genus can be quite ecologically dominant and conspicuous (see [7] for a review of the genus). The evolution of *Crematogaster* has recently been investigated in a molecular phylogenetic framework [9]. This study found that Madagascar was colonized by these ants through at least eight but possibly nine [10] independent dispersal events, producing six natural species-groups and three phylogenetically isolated species presently found on the island. Together these species-groups comprise 34 described and undescribed species of *Crematogaster* in Madagascar ([9], and unpubl. data).

In the present study we focus our attention on one of these species-groups, the *Crematogaster (Crematogaster) kelleri*-group (cf. [7], [9]). Prior to our work, three described species names (*C. adrepens* Forel, *C. kelleri* Forel and *C. madagascariensis* André) from Madagascar and one species name (*C. gibba* Emery) from the wider Malagasy region (the Seychelles) could be associated with this group. In addition, we here treat one of the above-mentioned phylogenetically isolated taxa, a relatively closely related and yet undescribed species. The *C. kelleri*-group is distributed throughout most Malagasy rainforests and dry forest habitats, but is absent from the arid southwest of the island. Most species in this group appear to nest arboreally in dead branches and under bark or canopy moss, although natural history data is sparse for less common taxa. On average, these ants are rather small and – contrary to the more usual habits of acrobat ants – not very conspicuous or aggressive (B. B. Blaimer, pers. observ.).

As in other species-groups [11], [12], the *Crematogaster kelleri*-group appears to contain one widespread and morphologically highly variable species, together with a series of more localized and less problematic species.

Here we therefore resolve and revise the taxonomy of the *C. kelleri*-group with a combination of morphological and genetic data, the latter comprising both mitochondrial and nuclear DNA. This revision represents the penultimate taxonomic treatment of the ecologically important acrobat ants in Madagascar (see [10]–[12]), and the results have a multitude of implications and applications in ecological and conservation research.

## Materials

### Specimen Field Collections

All necessary research and export permits for the field studies in Madagascar that generated the ant specimens used for morphological and molecular work in this study have been obtained from the following responsible Malagasy authorities: Madagascar National Parks (former ANGAP) and the Ministère de l’Environnement, et des Forêts.

### Morphological Study

Morphological observations were made with a Leica MZ9.5, MZ12.5 and MZ Apo stereomicroscope. Standard measurements (in mm) were taken at 16−50× with a Leica MZ Apo stereomicroscope and a dual-axis Acu-Rite Quikcount micrometer wired to a digital readout. Measurements are given to the second decimal place, indices are presented as decimal fractions (also to the second decimal) and ranges express minimum – maximum values. Measured specimens were chosen to represent the entire respective species distribution range and morphological variation. The abbreviations used for measurements and indices are described and illustrated in detail by Blaimer [10]–[12]. Selected measurements were visualized by creating scatter plots within Microsoft Excel.

Color images were created with a JVC KY-F75U digital camera, a Leica MZ16A stereomicroscope, Syncroscopy Auto-Montage (version 5.0) and ZERENE STACKER (v1.04) software. All ant images presented here are also publicly available on AntWeb (www.antweb.org). Species distributions were plotted with the packages “maps” and “mapdata” within the software R [13], based on collection coordinates (latitude and longitude) of all material examined (see Table S2 for a species list with GPS coordinates). For material lacking this primary information, i.e. syntype specimens and older material, the following sources were used to georeference collection sites: the GEOnet Names Server [14] and the Gazetteer to Malagasy Botanical Collecting Localities [15]. Maps of sampling localities were created with the module Cartographer [16] for the Mesquite system [17]. Classification of major geographic regions in Madagascar throughout species descriptions follows Gautier and Goodman [18]; common abbreviations of locality data use the acronyms P.N. ( = Parc National), R.S. ( = Réserve Spéciale), F ( = Forêt) and R.N.I.( = Réserve Naturelle Intégrale).

### Nomenclatural Acts

The electronic edition of this article conforms to the requirements of the amended International Code of Zoological Nomenclature [19], and hence the new names contained herein are available under that Code from the electronic edition of this article. This published work and the nomenclatural acts it contains have been registered in ZooBank, the online registration system for the ICZN. The ZooBank LSIDs (Life Science Identifiers) can be resolved and the associated information viewed through any standard web browser by appending the LSID to the prefix “http://zoobank.org/”. The LSID for this publication is: urn:lsid:zoobank.org:pub:B72DA7E7-8114-449C-B76B-C1BB7ADB97E9. The electronic edition of this work was published in a journal with an ISSN, and has been archived and is available from the following digital repositories: PubMed Central, LOCKSS.

The International Commission on Zoological Nomenclature requires lectotypes designated after 1999 to “contain an express statement of deliberate designation” (amended Article 74.7.3). We use the statement ‘lectotype by present designation’ to fulfill this requirement. Lectotypes have been designated where a name lacks a holotype or lectotype and unambiguous syntypes have been identified. The purpose is to provide stability of nomenclature, and designation is done in a revisionary context in agreement with the amended Recommendation 74G of Article 74.7.3.

### Specimens were Examined and/or Deposited in the Following Collections

**CASC** California Academy of Sciences, San Francisco, CA, USA

**BBBC** B.B. Blaimer Coll., Smithsonian Museum of Natural History, Washington, D.C., USA

**MCZC** Museum of Comparative Zoology, Harvard, USA

**MHNG** Muséum d’Histoire Naturelle, Genève, Switzerland

**MSNG** Museo Civico di Storia Naturale, Genova, Italy

**NHMB** Naturhistorisches Museum, Basel, Switzerland

**PSWC** P.S. Ward Collection, University of California at Davis, CA, USA

**SAMC** South African Museum, Cape Town, South Africa

**ZMHB** Museum für Naturkunde der Humboldt Universität, Berlin, Germany

### Molecular Data Collection and Phylogenetic Analyses

One to four individual worker ants for five of the six putative species treated in the present study were selected for molecular phylogenetic analysis. Sequence data could not be obtained from one species (*C. tavaratra*). From these 13 specimens, DNA was extracted from either worker adults or pupae using a DNeasy Tissue Kit (Qiagen Inc., Valencia, California, U.S.A.), following the manufacturer’s protocol but with final elution in sterilized water rather than the supplied buffer and at half the suggested volume. We used either a non-destructive method (cuticle of ant pierced prior to extraction, mostly used for adults) or a destructive technique (entire ant pulverized, mostly used for pupae) in cases where multiple individuals from the same colony series were available.

Three nuclear protein-coding genes were amplified: *long wavelength rhodopsin* (LW Rh, 856 bp exon/252 bp intron), *arginine kinase* (ArgK, 390 bp exon/147 bp intron) and *carbomoylphosphate synthase* (CAD, 568 bp exon/151 bp intron). The sequence lengths given here refer to the aligned sequence data included in phylogenetic inference and add up to a total of 2364 bp. The three amplified nuclear genes are widely used for phylogenetic inference in ants, primers are available [9], [20]–[22] and their usefulness in phylogenetic inference between closely related species has been demonstrated [10], [12], [23]. Amplifications were performed using standard PCR methods outlined in Ward and Downie [20] and sequencing reactions were analyzed on an ABI 3730 Capillary Electrophoresis Genetic Analyzer with ABI BigDye Terminator v3.1 Cycle Sequencing chemistry (Applied Biosystems Inc., Foster City, CA).

For separate mitochondrial genetic analysis, DNA was extracted destructively from the legs of a total of 64 specimens at the Biodiversity Institute of Ontario, University of Guelph (under the Barcode of Life Initiative), using extraction protocols as outlined in Smith & Fisher [24]. This dataset consists of one to 51 specimens for five of the six species treated here; DNA extraction and amplification was unsuccessful for one species (*C. hafahafa*). In general, the sample size, i.e. number of specimens, submitted for extraction to the Barcode of Life Initiative was much larger, but success rates were poor overall due to the advanced age of specimens. For this larger dataset, sequence data were collected for *cytochrome oxidase I* (COI, 578 bp used for inference) at the Biodiversity Institute of Ontario, with amplification and sequencing protocols as detailed in Smith & Fisher [24].

**Figure 1:**
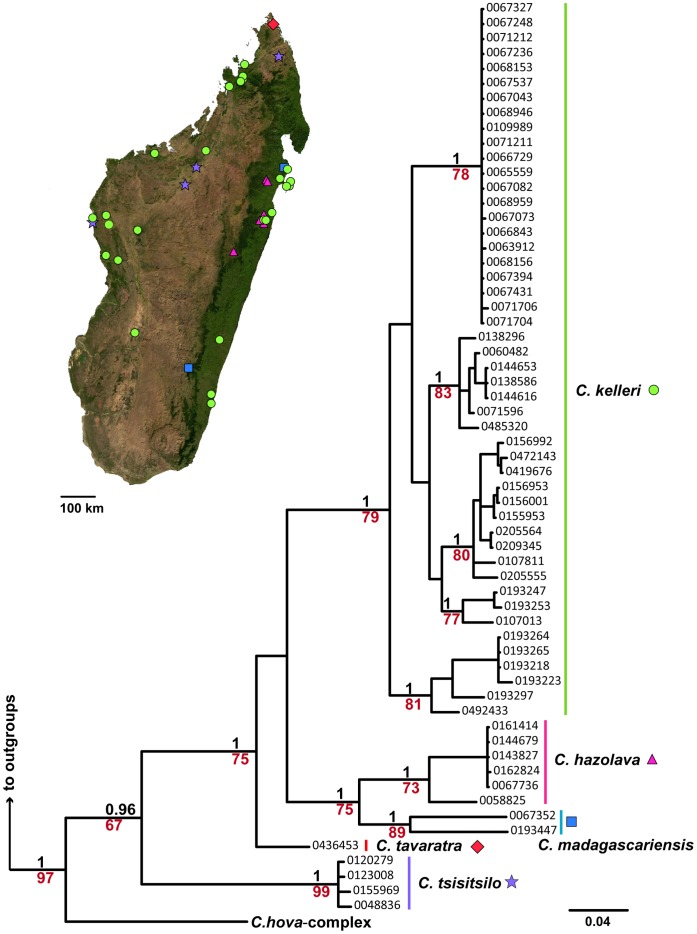
Mitochondrial phylogeny of the *C. kelleri*-group & related species. Results of Bayesian inference based on 578 bp of COI, summarized as a consensus tree in MrBayes. Support values in black represent posterior probabilities (pp), those in red ML bootstrap values, values pp<0.96 and bootstrap<60 are not shown; scalebar shows nucleotide changes per base pair. The long branch subtending outgroups *C. ranavalonae* and *C. madecassa* has been removed. Symbols beside species names on the phylogeny correspond to distribution markers in the adjacent map of Madagascar.

In a few cases both nuclear and mitochondrial data was obtained from extractions of the same specimen or colony series. Three Malagasy *Crematogaster* species from different species-groups were chosen as outgroups in both the nuclear and the mitochondrial dataset: *C. madecassa*, *C. ranavalonae* and the *C. hova*-complex. These range from moderately to distantly related to the focal group [9]. The nuclear sequences for these outgroups had already been published in prior studies [10], [12], the same is true for all or part of the sequence data for five of the ingroup taxa [9], [12], [21]. For the geographic distribution of the sampled taxa refer to Table S1 or Figure 1.

All newly generated sequences have been deposited in GenBank, and all accession numbers are listed in Table 1; data matrices and trees have further been deposited in TreeBase (ID13837; http://purl.org/phylo/treebase/phylows/study/TB2:S13837). Sequence data were assembled and edited in the program SEQUENCHER 5.0 (Gene Codes Corporation, 2006, Ann Arbor, MI) and aligned with MUSCLE [25], accessed through the CIPRES science gateway [26]. The nuclear data was concatenated into one single data matrix for which twelve data subsets were specified by gene, translational pattern (exon, intron) and codon position (1st, 2nd, 3rd). The COI dataset was subdivided into three data subsets according to codon positions. We then used PartitionFinder [27] to simultaneously choose the best-scoring partition scheme and to select best-fitting models of nucleotide sequence evolution for each data subset. Selected models for the chosen partition scheme can be found in Table 2.

**Table 1:** List of Genbank accessions.

nDNA	Voucher	LW Rh		ArgK	CAD			
***Crematogaster***								
*C. hafahafa*	CASENT0436545		KC514938	KC514925	KC514931			
*C. hazolava*	CASENT0058825		JQ326758	JQ326528	JQ326643			
*C. hazolava*	CASENT0151908		KC514936	KC514923	KC514929			
*C. hova*-complex	CASENT0058827		JQ326688	JQ326458	JQ326573			
*C. kelleri*	CASENT0498885		KC514940	KC544013	KC514933			
*C. kelleri*	CASENT0109989		JQ326746	JQ326516	JQ326631			
*C. kelleri*	CASENT0193255		KC514939	KC514926	KC514932			
*C. kelleri*	CASENT0071672		KC514941	KC514927	KC514934			
*C. kelleri*	CASENT0129802		KC514942	KC514928	KC514935			
*C. kelleri*	CASENT0193264		JN129957	JN129921	JN129850			
*C. madagascariensis*	CASENT0193446		KC514937	KC514924	KC514930			
*C. madagascariensis*	CASENT0193580		JQ326749	JQ326519	JQ326634			
*C. madecassa*	CASENT0068164		JQ326949	JQ326913	JQ326932			
*C. ranavalonae*	CASENT0193425		JN129942	JN129891	JN129871			
*C. tsisitsilo*	CASENT0155969		KC526924	KC526922	KC526923			
*C. tsisitsilo*	CASENT0120279		JQ326754	JQ326524	JQ326639			
**mtDNA**	**Voucher**		**COI**		**Voucher**		**COI**	
*C. hazolava*	CASENT0143827		HQ925531	*C. kelleri*	CASENT0193264		HQ547829	
*C. hazolava*	CASENT0144679		HQ925536	*C. kelleri*	CASENT0193223		HQ547824	
*C. hazolava*	CASENT0162824		JN283308	*C. kelleri*	CASENT0472143		HM418776	
*C. hazolava*	CASENT0067736		KC501950	*C. kelleri*	CASENT0067327		KC501939	
*C. hazolava*	CASENT0058825		HM880703	*C. kelleri*	CASENT0193297		HQ547837	
*C. hazolava*	CASENT0161414		JN283245	*C. kelleri*	CASENT0067236		KC501937	
*C. hova-complex*	CASENT0193380		HQ547857	*C. kelleri*	CASENT0209345		KC501938	
*C. madagascariensis*	CASENT0067352		KC501953	*C. kelleri*	CASENT0067073		KC501936	
*C. madagascariensis*	CASENT0193447		HQ547866	*C. kelleri*	CASENT0067248		KC501934	
*C. madecassa*	CASENT0068164		KC501955	*C. kelleri*	CASENT0067082		KC501935	
*C. ranavalonae*	CASENT0193531		HQ547877	*C. kelleri*	CASENT0068153		KC501933	
*C. tavaratra*	CASENT0436453		HM880707	*C. kelleri*	CASENT0068156		KC501932	
*C. tsisitsilo*	CASENT0155969		KC501952	*C. kelleri*	CASENT0067394		KC501942	
*C. tsisitsilo*	CASENT0048836		HM418724	*C. kelleri*	CASENT0066729		KC501931	
*C. tsisitsilo*	CASENT0123008		HM418747	*C. kelleri*	CASENT0066843		KC501949	
*C. tsisitsilo*	CASENT0120279		HM418732	*C. kelleri*	CASENT0065559		KC501930	
*C. kelleri*	CASENT0205555		KC501956	*C. kelleri*	CASENT0156992		JN283154	
*C. kelleri*	CASENT0193265		HQ547830	*C. kelleri*	CASENT0193253		HQ547827	
*C. kelleri*	CASENT0155953		JN283108	*C. kelleri*	CASENT0071704		KC501941	
*C. kelleri*	CASENT0156953		JN283150	*C. kelleri*	CASENT0068946		KC501929	
*C. kelleri*	CASENT0060482		KC501951	*C. kelleri*	CASENT0138586		HQ925525	
*C. kelleri*	CASENT0156001		JN283112	*C. kelleri*	CASENT0068959		KC501928	
*C. kelleri*	CASENT0067043		KC501940	*C. kelleri*	CASENT0205564		KC501927	
*C. kelleri*	CASENT0138296		HM418769	*C. kelleri*	CASENT0419676		HM879916	
*C. kelleri*	CASENT0144616		HQ925534	*C. kelleri*	CASENT0067431		KC501947	
*C. kelleri*	CASENT0193218		HQ547823	*C. kelleri*	CASENT0071212		KC501948	
*C. kelleri*	CASENT0485320		HM418781	*C. kelleri*	CASENT0071706		KC501926	
*C. kelleri*	CASENT0107013		DQ176164	*C. kelleri*	CASENT0492433		HM418785	
*C. kelleri*	CASENT0071211		KC501946	*C. kelleri*	CASENT0071596		KC501943	
*C. kelleri*	CASENT0109989		KC501954	*C. kelleri*	CASENT0107811		KC501944	
*C. kelleri*	CASENT0144653		HQ925535	*C. kelleri*	CASENT0067537		KC501925	
*C. kelleri*	CASENT0063912		KC501945	*C. kelleri*	CASENT0193247		HQ547826	

Genbank accessions of sequences included in this study. LW Rh: Long wavelength rhodopsin, ArgK: Arginine kinase, CAD: Carbomoylphosphate synthase, COI: Cytochrome oxidase I.

**Table 2:** Characteristics of data subsets and selected substitution models.

Data partition	No. bases	No. VC	No. PIC	Substitution model
*nDNA*				
LW Rh, ArgK and CAD, exon positions 1+2	1208	30 [2]	11 [9]	HKY+I
LW Rh and ArgK, exon positions 3	416	48 [10]	21 [12]	GTR+I
CAD, exon positions 3 and LW Rh introns	442	55 [11]	32 [22]	HKY+G
ArgK and CAD introns	298	46 [6]	11 [8]	HKY
**Total**	**2364**	**179 ** [**29**]	**75 [51]**	
*mtDNA*				
COI positions 1+2	385	19 [7]	41 [34]	HKY+I+G
COI positions 3	193	19 [14]	159 [150]	GTR+G
**Total**	**578**	**38 ** [**21**]	**200 [184]**	

Data subsets used in phylogenetic analyses and their characteristics; VC = variable characters, PIC = parsimony informative characters; [ ] = ingroup only. The partitioning scheme and the respective substitution models were chosen with the software PartitionFinder [27].

Phylogenetic analyses were performed on both nuclear and mitochondrial datasets within a Bayesian framework (BI hereafter) using MRBAYES v3.1 [28] via the California Academy of Sciences’s CCG Phylocluster, and within a maximum likelihood framework (ML hereafter) using GARLI v2.0 [29], accessed through the GARLI Web Service (http://molecularevolution.org). BI-analyses each employed two runs of Metropolis-coupled Markov Chain Monte Carlo (MCMCMC) consisting of four chains (temp = 0.05) and sampling every 1000 generations. The model parameters transition-transversion ratio, gamma shape, proportion of invariable sites, rate matrix and state frequencies were unlinked across partitions. A variable rateprior was employed during analysis of the nuclear dataset; for analysis of the COI dataset a dirichlet rateprior (*1,5*) was used and the branchlength prior settings were modified (*unconstrained:exponential (100)*) to obtain realistic values for rate multipliers and tree length. Convergence of chains and other diagnostic values were assessed in several ways. In MRBAYES we confirmed that the ASDSF had reached values well below 0.01 and PSRF values had approached 1.0 for all parameters. In TRACER v1.5 [30], convergence was confirmed visually and mixing of chains was evaluated with effective sample size (ESS) values. To assess whether tree topologies were sampled in proportion to their true posterior distribution, we further used the compare, slide and cumulative plotting functions on the AWTY-online server [31]. All the above indicators returned good values after MCMCMC-sampling for 20 million generations; consensus trees were summarized in MRBAYES after discarding 25% of samples as burnin. A ML bootstrap search with 1000 replicates was performed in GARLI, with the resulting trees summarized as a majority-rule consensus tree. All trees were rooted with the outgroup method using the most distantly related taxon, *Crematogaster madecassa* (see [9]).

Mean, minimum and maximum sequence divergences of COI within and between species were calculated under the Tamura-Nei model [32] with the software MEGA5 [33].

## Results

### Taxonomic Rationale

Both nuclear and mitochondrial sequence data corroborate the a priori morphological distinction of five species within the *Crematogaster kelleri*-group: the previously described *C. kelleri* Forel and *C. madagascariensis* André, and three new species, *C. hazolava* sp. n., *C. hafahafa* sp. n. and *C. tavaratra* sp. n. Further supported is one new species outside of this species-group, *C. tsisitsilo* sp.n. Our data, both morphological and molecular, do not support the previously described *C. adrepens* Forel and *C. gibba* Emery as distinct species. We were able to morphologically match the syntype material of these taxa to recent collections, but found these to be not significantly distinct from the *C. kelleri* type material and other examined specimens. *Crematogaster adrepens* and *C. gibba* are therefore synonymized in the following treatment.

### Phylogenetic Results

**Figure 2:**
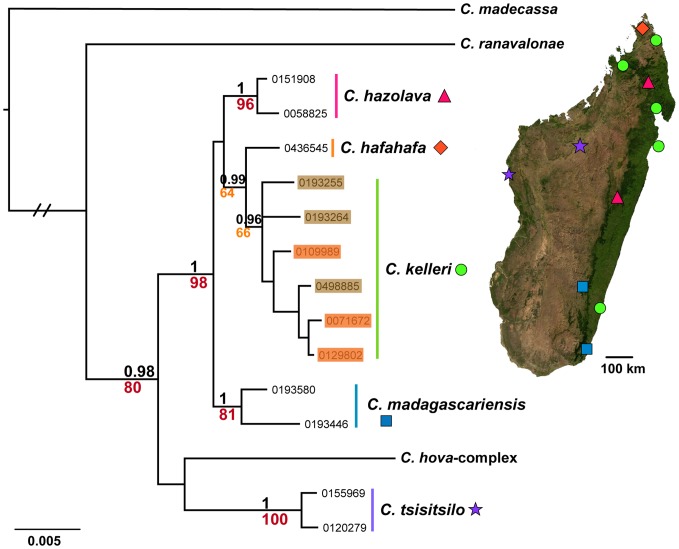
Nuclear phylogeny of the *C. kelleri*-group and related species. Results of Bayesian inference based on 2364 bp of the three nuclear genes LW Rh, ArgK and CAD, summarized as consensus tree in MrBayes. Support values in black represent posterior probabilities (pp), those in red or orange ML bootstrap values, values pp<0.96 and bootstrap<60 are not shown; scalebar shows nucleotide changes per base pair. Yellow and brown colored form of *C. kelleri* are indicated by orange and brown shading, respectively. The long branch subtending outgroups *C. ranavalonae* and *C. madecassa* has been shortened. Symbols beside species names on the phylogeny correspond to the distribution markers in the adjacent map of Madagascar.

The molecular phylogenetic results of BI and ML analyses do not vary significantly in topology, therefore ML bootstrap results are shown summarized on the BI topology for both COI (Fig. 1) and nuclear data (Fig. 2). Newly defined species are indicated on the phylogenies, with the respective sampling localities indicated on the adjacent maps. *Crematogaster tavaratra* is absent from the mitochondrial phylogeny (Fig. 1), while *C. hafahafa* is absent from the nuclear phylogeny (Fig. 2). All other species are well supported by the mitochondrial data with posterior probabilities (PP) = 1 and bootstrap values >70. Molecular support by the nuclear data is also strong overall. Exceptions to this rule are the lower values (PP = 0.96, bootstrap = 66) associated with *C. kelleri*, which could be an artifact of including only a single sequence of its suggested sister taxon, *C. hafahafa*, in the analysis.

Both nuclear and CO1 data lend firm phylogenetic support for the *C. kelleri* species-group (Fig. 2 and Fig. 1, respectively). In the larger phylogenetic analysis of Malagasy *Crematogaster* [9], *C. tsisitsilo* was shown to be distantly related and not part of the *C. kelleri*-group. Here, the nuclear phylogeny indicates the same result, albeit with weak support. The opposite conclusion is suggested by the mitochondrial phylogeny, which recovers *C. tsisitsilo* as sister group to the *C. kelleri*-group. Both nuclear data sets analyzed here and in the larger study [9] did not include *C. tavaratra*. This species could be crucial, however, for a well-supported estimation of these relationships, since it is indicated as the basal lineage within the *C. kelleri*-group in the mitochondrial analysis. The closest affinities of *C. tsisitsilo* therefore remain unclear. Relationships within the *C. kelleri*-group are also inconclusive due to the two missing taxa in nuclear and mitochondrial phylogenies (*C. tavaratra* and *C. hafahafa*, respectively).

### Molecular and Morphological Variation

**Figure 3:**
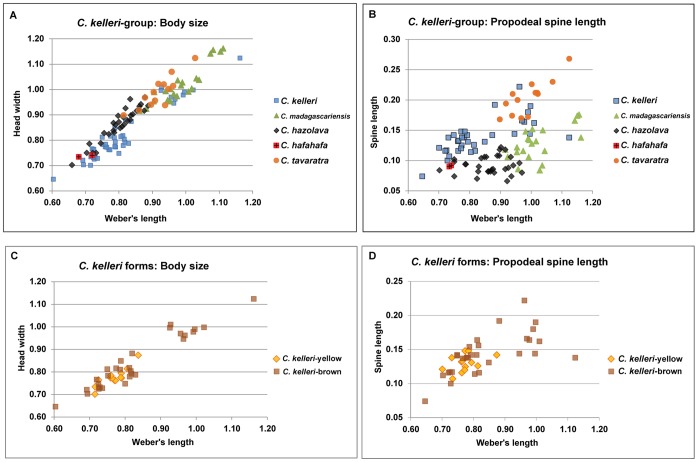
Graphical plots of selected measurements of species within the *C. kelleri*-group. Based on specimen measurements as presented within species descriptions, units in mm. **A**: body size as a function of head width over Weber’s length in the *C. kelleri*-group; **B:** Propodeal spine length as a function of spine length over Weber’s length in the *C. kelleri*-group; **C**: body size as a function of head width over Weber’s length in the two forms of *C. kelleri*; **D**: Propodeal spine length as a function of spine length over Weber’s length in the two forms of *C. kelleri*.

**Figure 4:**
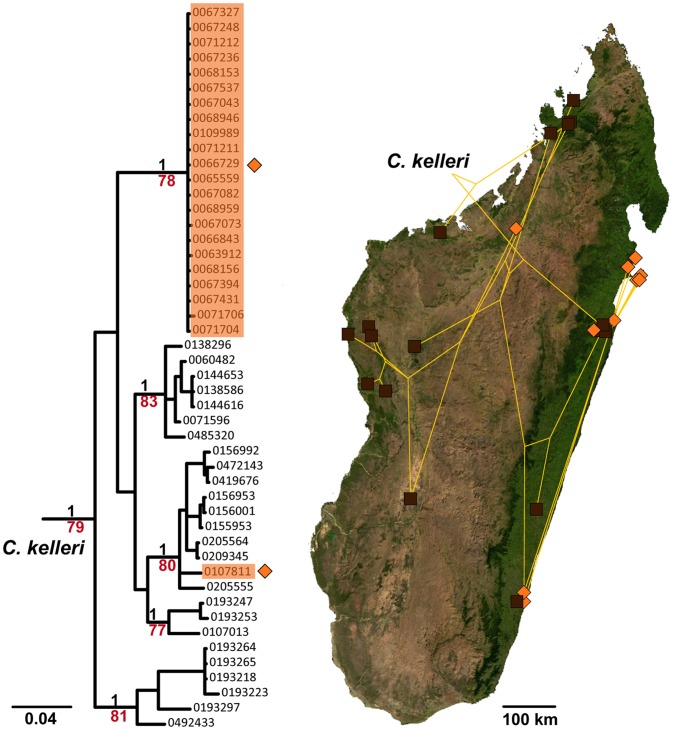
Mitochondrial phylogeny of *C. kelleri* with respect to variation and distribution data. Results of Bayesian inference based on 578 bp of COI as shown in Fig. 1, but showing only the *C. kelleri* clade. Support values in black represent posterior probabilities (pp), those in red ML bootstrap values, values pp<0.96 and bootstrap<60 are not shown; scalebar shows nucleotide changes per base pair. On the phylogeny, orange-shaded specimens indicate the yellow form of *C. kelleri*, unshaded specimens belong to the brown form. On the map of Madagascar, the yellow form is indicated by orange diamonds and the brown form by squares; the lines connecting the markers represent the phylogeny as plotted on the map with CARTOGRAPHER [16].

We provide plots of body size and propodeal spine length for all taxa in the *C. kelleri-*group (Fig. 3A−D) to illustrate that these measurements can partly, or in some cases completely, separate species within this group. In particular *Crematogaster kelleri* was found to be a morphologically highly variable taxon (see also species description), thus an effort was made to investigate the full breadth of this morphological variation with molecular methods. The mtDNA phylogeny was further mapped onto the sampling distribution map (Fig. 4) to discern whether observed molecular and morphological variation in *C. kelleri* corresponds with geography.

While characters such as body size (Fig. 3A) vary continuously within this species, *C. kelleri* also shows an interesting discontinuous variation in color. One form is variably brown colored (all shades), whereas the other is yellow. Morphologically, the brown form is far more variable than the yellow form (Fig. 3C,D), yet the forms occur to some extent in sympatry. We thus investigated the possibility that these two forms of *C. kelleri* actually belong to two closely related sister species. The two color forms are indicated on the nuclear (Fig. 2) and the COI phylogeny (Fig. 4), and separate distribution maps have been created (see species descriptions). The results suggest that the yellow form tends to be prevalent in the coastal regions of eastern, northern and north-western Madagascar, but that the two forms are not genetically isolated from one another (Fig. 2 and 4). Although there is a large, entirely ‘yellow clade’ in the COI phylogeny (Fig. 4), this appears to be an artifact of geography as samples here all stem from east coast localities. Only one specimen of the yellow form from north-western Madagascar was successfully sequenced and was found to be more closely related to other western (brown) populations than to eastern yellow populations. However, the low sequence divergence in this east coast ‘yellow clade’ is peculiar and requires further investigation.

*Cytochrome oxidase I* sequence divergences are summarized within and between species of the *C. kelleri*-group and *C. tsisitsilo* in Table 3. Within-species sequence divergences range from 0−14.1%, with the greatest sequence variation within *C. kelleri* and *C. madagascariensis*. Mean sequence divergences between species of the *C. kelleri*-group range from 15.3−20.2%, and divergences of the *C. kelleri*-group to *C. tsisitsilo* range between 19.1% (to *C. tavaratra*) and 25.1% (to *C. hazolava*).

**Table 3:** COI sequence divergences within and between species of this study.

	within species			between species (mean)			
	mean	min.	max.	*C. kelleri*	*C. hazolava*	*C. madagascariensis*	*C. tavaratra*
*C. kelleri*	0.068	0.000	**0.140**				
*C. hazolava*	0.030	0.000	0.085	0.190			
*C. madagascariensis*	**0.141**	n/a	n/a	**0.202**	0.174		
*C. tavaratra*	n/a	n/a	n/a	0.153	0.158	0.175	
*C. tsisitsilo*	0.014	0.008	0.017	0.210	**0.251**	0.236	0.191

Divergences of *cytochrome oxidase I* sequences, as estimated under the Tamura-Nei Model [32] within the software MEGA5 [33]. Bold font emphasizes highest levels of divergence within species, between species, and between *C. tsisitsilo* and the *C. kelleri*-group.

### Checklist of Species Treated in this Revision

*Crematogaster tsisitsilo* **sp. nov.**

***Crematogaster kelleri*** **-group:**

*Crematogaster kelleri* Forel 1891

 = *Crematogaster adrepens* 1897 **syn. nov.**

 = *Crematogaster gibba* Emery 1894 **syn. nov.**

*Crematogaster madagascariensis* André 1887

*Crematogaster hazolava* **sp. nov.**

*Crematogaster tavaratra* **sp. nov.**

*Crematogaster hafahafa* **sp. nov.**

### Diagnoses of the *Crematogaster kelleri*-group

#### Worker diagnosis of the Crematogaster kelleri-group

C. kelleri, C. madagascariensis, C. hafahafa, C. hazolava, C. tavaratra. Very small to large size (HW 0.60–1.16, WL 0.65–1.16).

Masticatory margin of mandibles with four teeth (five teeth in some large specimens of C. kelleri). Head shape fairly quadrate, usually wider than long (CI 1.04–1.16); posterior margin of head in full face view laterally rounded or subangular, sometimes medially slightly depressed; occipital carinae present; antennal scapes usually easily surpassing head margin, sometimes merely reaching head margin; midline of eyes situated at or slightly above midline of head in full face view; eyes fairly large (OI 0.18–0.27) and moderately protruding.

Pronotum laterally subangular; promesonotal suture usually absent or indistinct; mesonotum usually with a distinct dorsal and posterior face, and laterally carinate, angulate or subangulate; mesonotum often with posterolateral angular tubercules or denticles, and in lateral view outline of promesonotum usually characteristic: pronotum and dorsal face of mesonotum form a straight or slightly rounded plane, while the posterior face of the mesonotum slopes abruptly and steeply into the metanotal groove; metanotal groove shallow; propodeal spines very short to medium-sized (SPI 0.07–0.27), spiniform; dorsal face of propodeum almost as long as posterior face; petiole in dorsal view oval or suboval, rarely moderately flared, subpetiolar process variable from absent to a reduced angular dent to a small, but distinct tooth; postpetiole bilobed, but depth of median impression variable; subpostpetiolar process absent.

Sculpture reduced overall; head shiny to aciculate; mesosoma dorsally shiny and aciculate; propleuron aciculate, meso- and metapleuron mostly reticulate, sometimes carinulate; dorsal face of propodeum aciculate, posterior face shiny; dorsal face of petiole shiny; helcium dorsally carinulate; postpetiole dorsally aciculate; lateral and ventral face of petiole and postpetiole feebly reticulate; face with 4–14 (more commonly 8–12) erect, long, flexuous setae, and abundant shorter, decumbent to subdecumbent pubescence; number of erect, long, flexuous setae on promesonotum highly variable, but usually present are at least four pronotal setae, and two lateral setae each on dorsal and posterior face of mesonotum; petiole with a pair of long, erect setae on dorsoposterior tubercules; postpetiole with at least one pair of long erect dorsoposterior setae, sometimes additional erect pilosity; abdominal tergites and sternites four to seven with long erect pilosity that is mostly abundant though highly variable, and abundant to very abundant appressed, decumbent or suberect pubescence throughout. Color pale to medium yellow, light to dark brown.

#### Queen diagnosis of the Crematogaster kelleri-group

*C. tavaratra, C. hazolava, C. kelleri*, *C. madagascariensis*. Small to medium size (HW 1.15–1.45, WL 1.70–2.34); with worker characters, except as follows.

Masticatory margin of mandibles with five to six teeth; antennal scapes of variable length; eyes fairly large (OI 0.27–0.32) and protruding, situated at or below midline of head in full face view; head shape quadrate, wider than long (CI 1.05–1.20), widest just posterior to eyes; posterior margin of head straight.

Mesosoma fairly compact (MSNI 0.75–0.98, WL 1.70–2.34); mesoscutum in dorsal view varying from longer than wide to as wide as long; dorsal face of propodeum short, posterior face abruptly sloping; propodeal spines present or absent (SPI 0.00–0.10), spiniform or dentiform; petiole lacking dorsoposterior tubercules; postpetiole with worker characters, but median impression less pronounced.

Sculpture mostly shiny and aciculate except for metapleuron, and with anteriormost part of propodeum carinulate, and face between eyes and antennal insertions sometimes costulate; erect pilosity moderately abundant on head, dorsal side of mesosoma and on metasoma, but shorter than in workers; petiole with or without a pair of long, flexuous dorsoposterior setae, sometimes other erect setae present laterally; erect pilosity on postpetiole variable, usually with at least one pair of dorsoposterior setae. Color similar to respective workers, but metasoma often darker. Wings clear.

#### Male diagnosis of the Crematogaster kelleri-group

C. tavaratra, C. hazolava, C. kelleri, C. madagascariensis.

Very small to small (HW 0.47–0.65, WL 0.85–1.24). Masticatory margin of mandibles with two to three teeth; clypeus squarely protruding; eyes large (OI 0.40–0.68) and protruding, midline of eyes situated well below midline of head, approaching clypeal margin; antennae 12-segmented, scapes very short (SI 0.18–0.24); head wider than long (CI 1.17–1.35); in full face view ocellar triangle situated at or below posterior head margin and may be slightly elevated with respect to rest of face; occipital carinae very distinct.

Mesosoma compact (MSNI 0.84–1.39, WL 0.85–1.24); mesoscutum in dorsal view as wide as long; scutellum in dorsal view laterally compressed, often distinctly tapering from anterior to posterior end, dorsoposterior portion pointed or truncate; dorsal face of propodeum very short or absent; propodeal spines absent; petiole in dorsal view oval, carinae or denticles absent and all margins rounded, in lateral view petiole anteriorly greatly tapered; subpetiolar process absent; postpetiole with weak median impression, more pronounced posteriorly; wings clear.

Head sculpture shiny to rugulose; clypeus with several carinulae; mesoscutum rugulose, scutellum shiny to aciculate, propodeum longitudinally carinulate, petiole and postpetiole rugulose to shiny; erect pilosity on face variable; mesoscutum with regular or scattered short, erect pilosity; posterior part of scutellum with more abundant, longer pilosity; petiole and postpetiole with distinct long dorsoposterior setae, and abundant erect pilosity dorsally and laterally; abdominal segments four to seven with abundant short suberect pubescence, longer erect pilosity lacking. Color mostly as in worker and queen, sometimes paler.

In the individual species descriptions below, only refinements or characters in addition to the above will be mentioned.

### Short Diagnosis of the *C. kelleri*-group and *C. tsisitsilo*

From all other Malagasy *Crematogaster* the species treated in this revision can be distinguished by the following characters; note that a key to all the species-groups of *Crematogaster* in Madagascar is currently under way (Blaimer & Fisher, in prep.).

#### Crematogaster kelleri-group

1) In lateral view promesonotum forming a straight (or at most slightly rounded) plane; 2) posterior face of the mesonotum long and distinctly set off from dorsal face and abruptly and steeply sloping into metanotal groove; 3) propodeal spines always spiniform, very short to medium-sized (SPI 0.07–0.24) and situated high on propodeum.

#### Crematogaster tsisitsilo

1) propodeal spines absent; 2) mesonotum greatly raised with respect to pronotum and propodeum; 3) eyes large (OI ≥0.21); 4) color orange or brown.

### Key to the Workers of the *C. kelleri*-group

**Figure 5:**
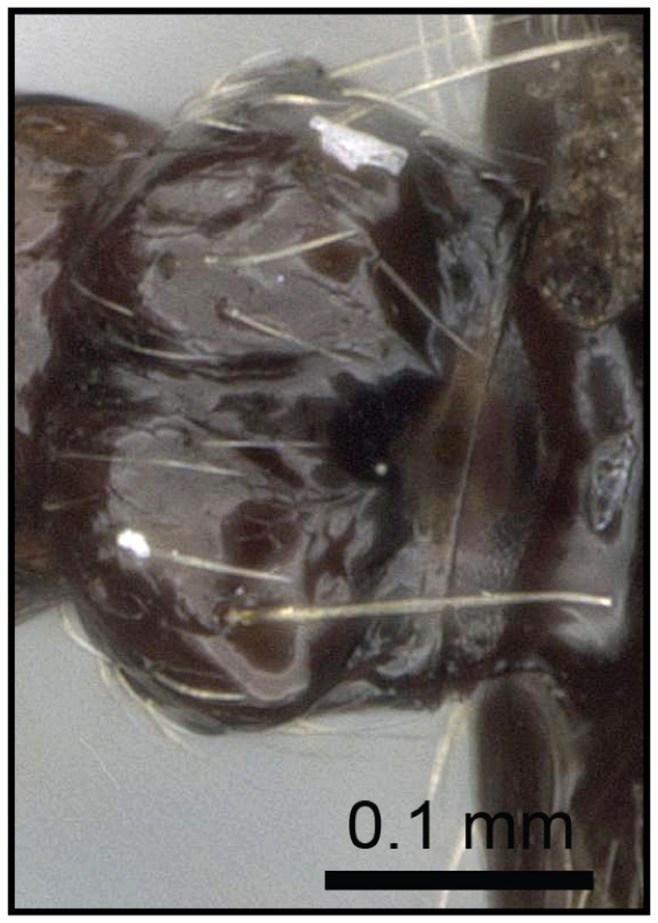
Species-key to the *C. kelleri*-group. Postpetiole with a complete, deep, longitudinal median impression (*C. madagascariensis*, CASENT0193446).

**Figure 6:**
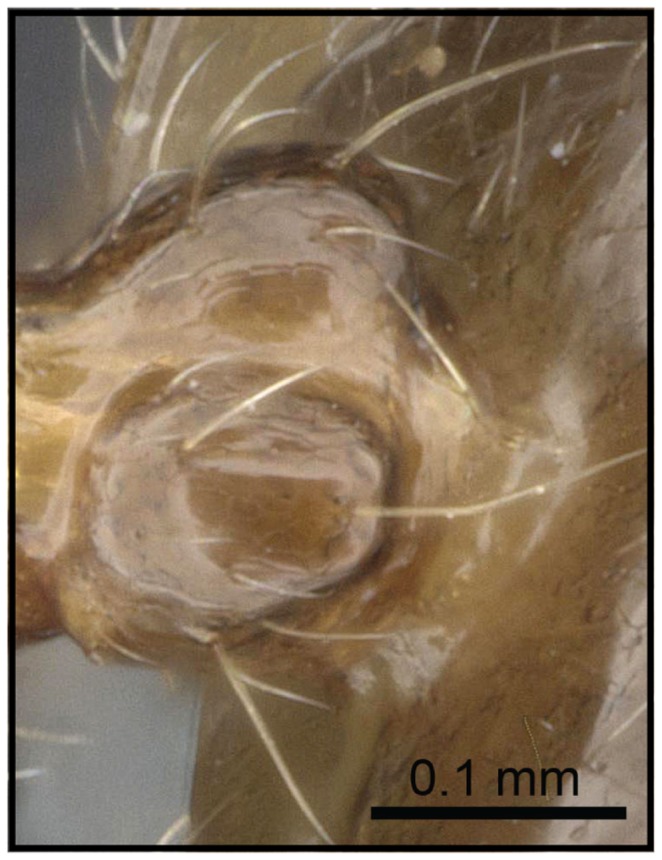
Species-key to the *C. kelleri*-group. Postpetiole with median stripe on complete median impression (*C. hazolava*, CASENT0317642).

**Figure 7:**
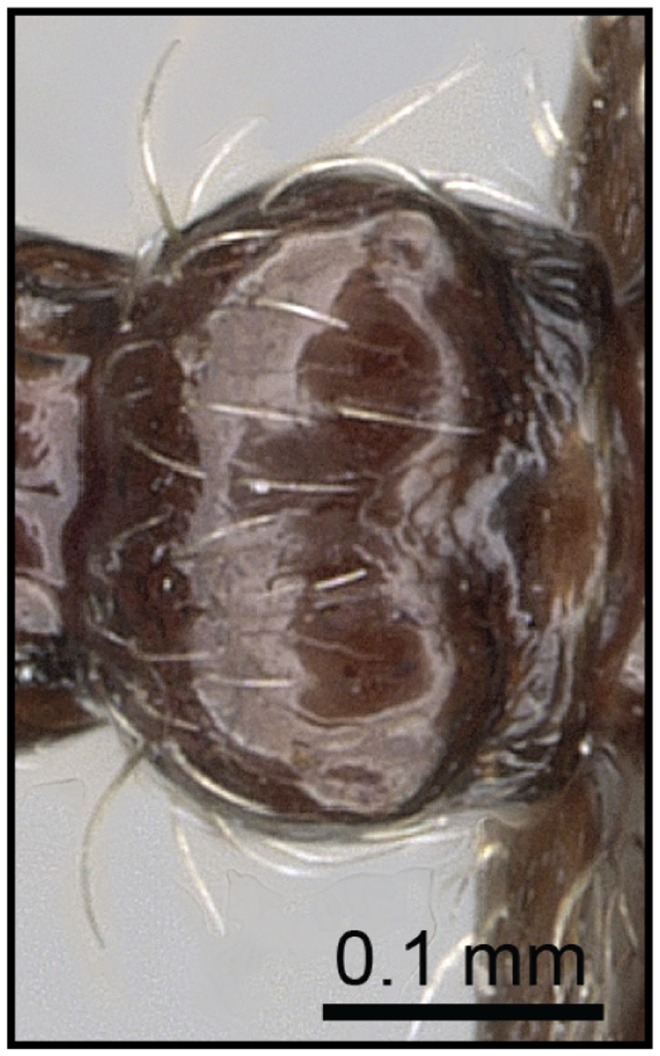
Species-key to the *C. kelleri*-group. Postpetiole with shallow median impression (*C. kelleri*, CASENT0454305).

**Figure 8:**
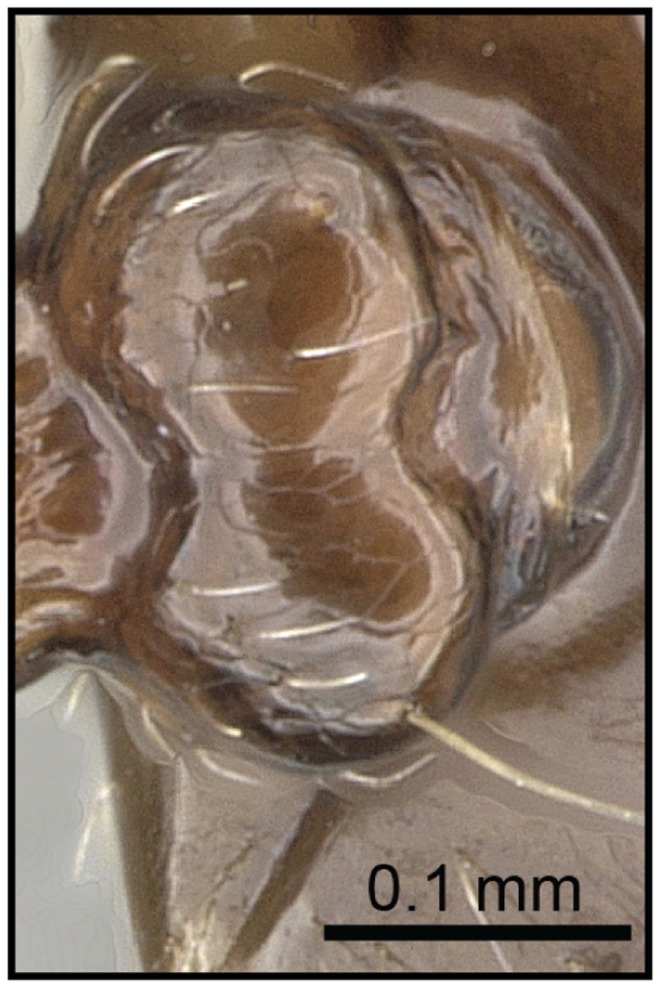
Species-key to the *C. kelleri*-group. Postpetiole with shallow median impression (*C. kelleri*, CASENT0466090).

**Figure 9:**
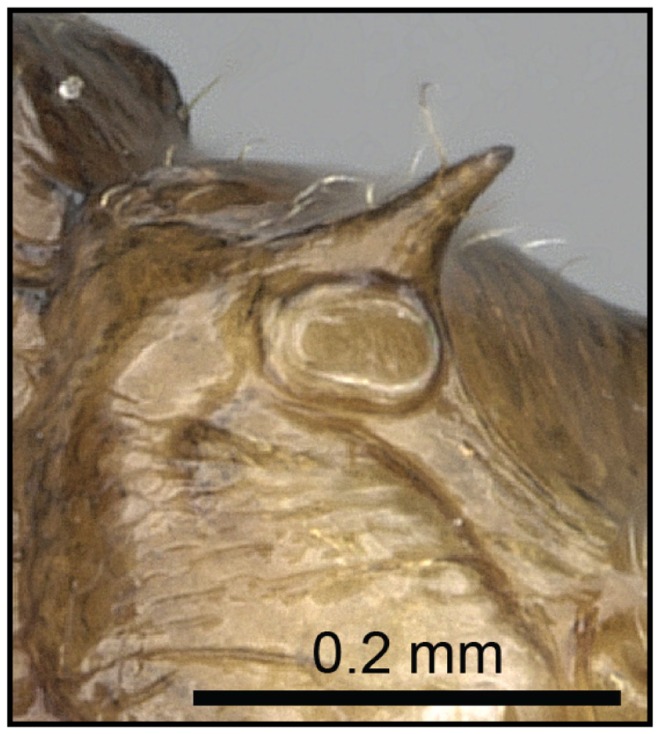
Species-key to the *C. kelleri*-group. Propodeal spiracle confluent with base of propodeal spines (*C. hazolava*, CASENT0317642).

**Figure 10:**
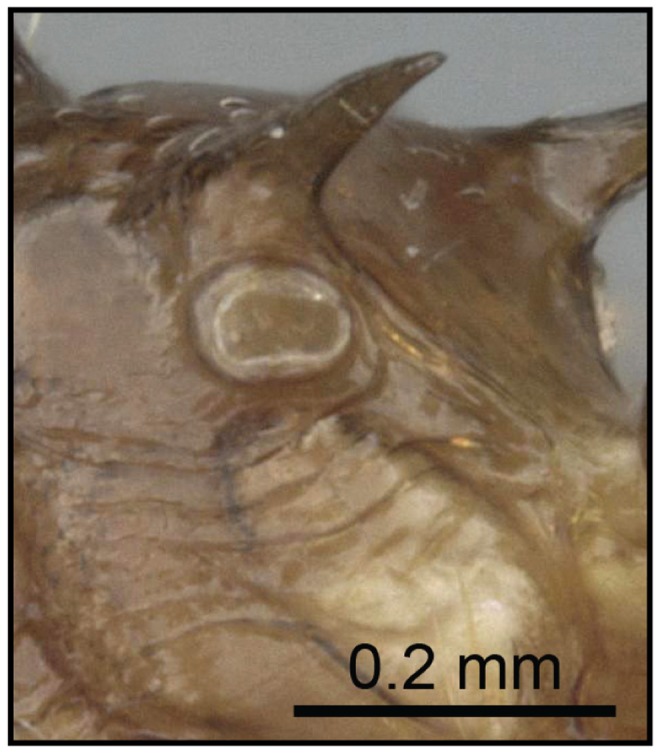
Species-key to the *C. kelleri*-group. Propodeal spiracle situated below base of propodeal spines (*C. tavaratra*, CASENT0436456).

- 1 Postpetiole clearly bisected by a complete, deep, longitudinal median impression (Figs 5, 6)…. **2**
  – Postpetiole with shallow median impression anteriorly, more pronounced posteriorly (Figs 7, 8) ….***C. kelleri***
- 2 (1) Propodeal spiracle mostly confluent with base of propodeal spines (Fig. 9); propodeal spines short (SPI 0.07–0.15)…… **3**
  - Propodeal spiracle situated below base of propodeal spines (Fig. 10); propodeal spines medium length (SPI 0.17–0.24); *far northern Madagascar* ……***C*** **.** ***tavaratra***
- 3 (2) Postpetiole evenly colored (as in Fig. 5, 7, 8), body color other than yellow……**4**
  – Postpetiole with “median stripe”: longitudinal impression lighter colored than lobes (as in Fig. 6), or body color yellow (in this case median stripe present, but hard to see).…***C.*** ** ***hazolava***
- 4 (3) Very small species (HW 0.68–0.72, WL 0.73–0.74); posterior head margin rounded; color dirty yellow; *known only from Montagne d’Ambre massif*……***C. hafahafa***
  – Medium to large species (HW 0.86–1.11, WL 0.92–1.16); posterior head margin usually with subangular corners, color brown or dark brown ….***C. madagascariensis***

### Key to the Queens of the *C. kelleri*-group (*C. tavaratra, C. hazolava, C. kelleri, C. madagascariensis*)

**Figure 11:**
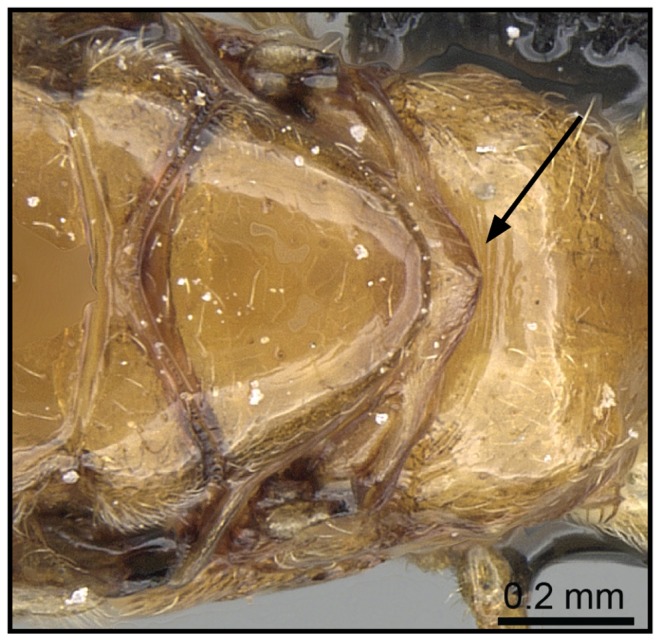
Species-key to the *C. kelleri*-group. Metanotum projecting from below scutellum in dorsal view (*C. kelleri*, CASENT0124998).

**Figure 12:**
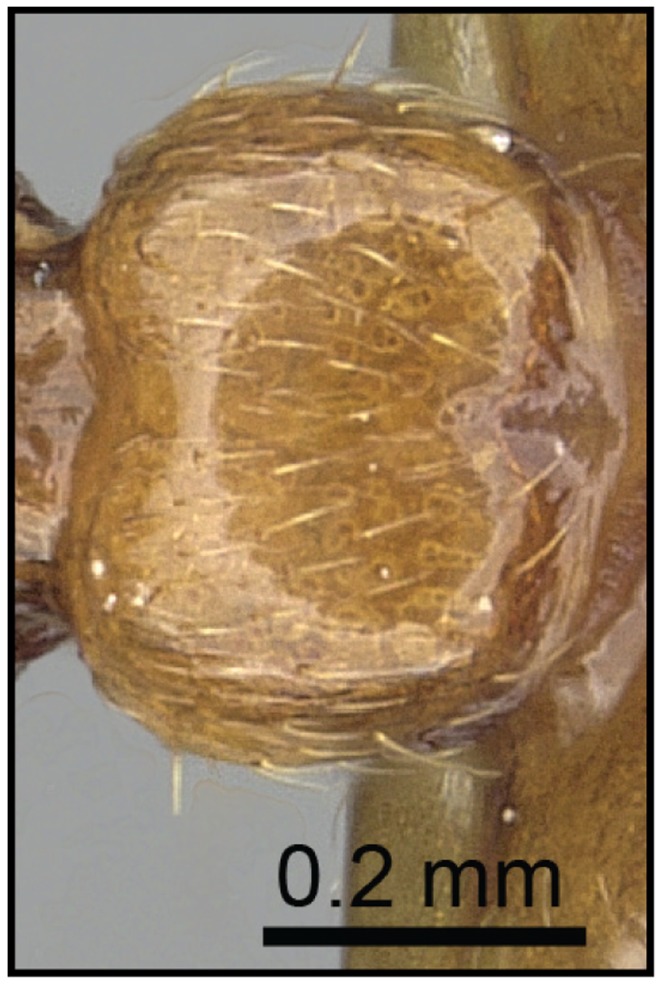
Species-key to the *C. kelleri*-group. Postpetiole with superficial impression (*C. kelleri*, CASENT0124998).

**Figure 13:**
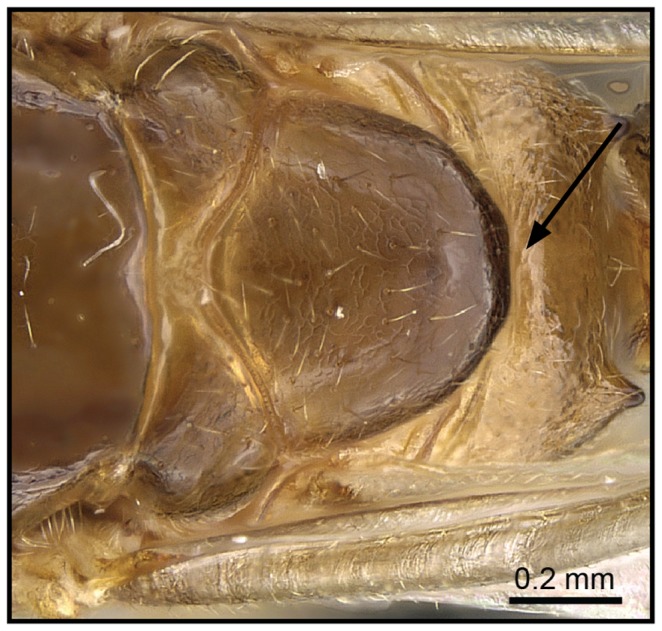
Species-key to the *C. kelleri*-group. Metanotum not projecting in dorsal view, covered by scutellum (*C. hazolava*, CASENT0160002).

**Figure 14:**
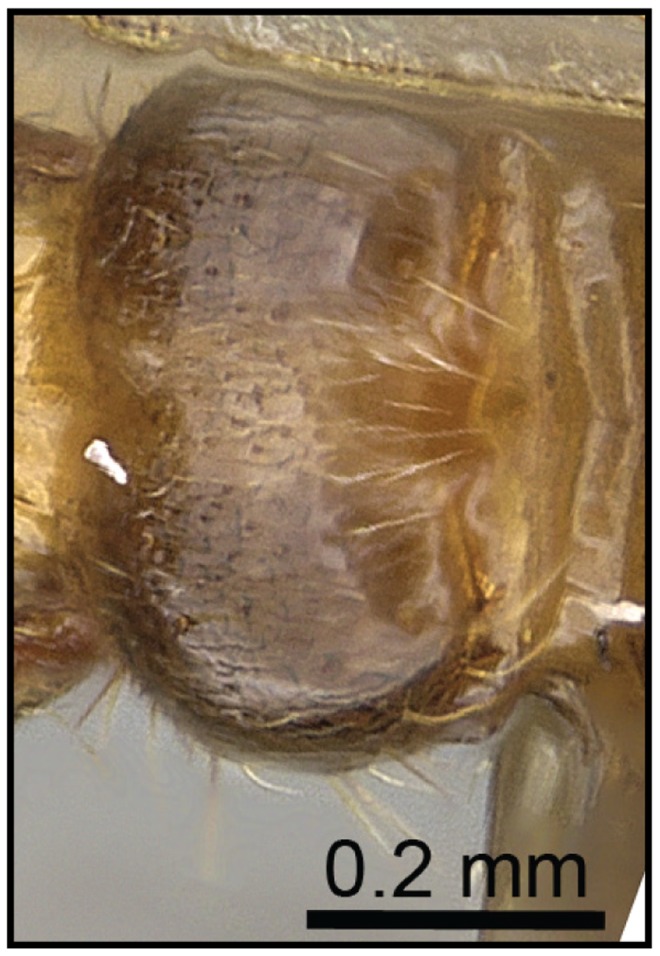
Species-key to the *C. kelleri*-group. Postpetiole with median stripe on longitudinal impression (*C. hazolava*, CASENT0160002).

- 1 Propodeal spines short, or reduced to dents or indistinct tubercules (SPI 0.00–0.07); scapes often not reaching head margin (SI 0.65–0.74)….**2**
  – Propodeal spines distinctly spiniform (SPI 0.09–0.10); scapes reaching or surpassing head margin (SI 0.72–0.77)….***C.*** ** ***tavaratra***
- 2 (1) Metanotum projecting from below scutellum in dorsal (Fig. 11) and lateral view; postpetiole merely with superficial impression (Fig. 12)….***C. kelleri***
  – Metanotum not projecting, covered by scutellum (Fig. 13); postpetiole with shallow but distinct longitudinal impression (Fig. 14) …. **3**
- 3 (2) Postpetiole with “median stripe” on longitudinal impression (Fig. 14)…. ***C. hazolava***
  – Postpetiole uniformly colored, without median stripe on longitudinal impression…. ***C. madagascariensis***

### Key to the Males of the *C. kelleri*-group (*C. tavaratra, C. hazolava, C. kelleri, C. madagascariensis*)

**Figure 15:**
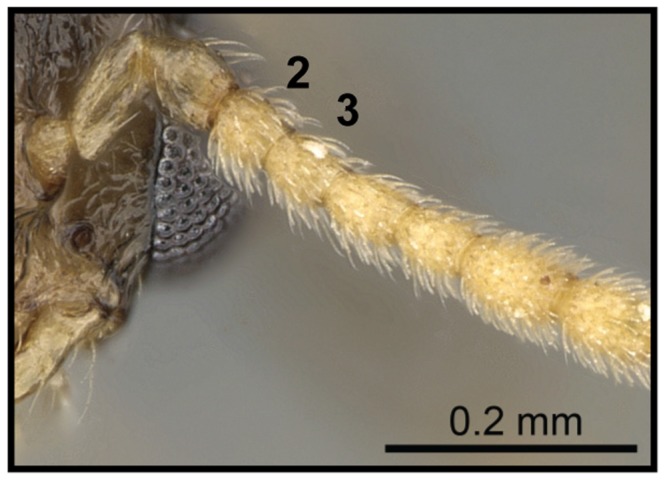
Species-key to the *C. kelleri*-group. Antennae with second and third funicular segment longer than wide (*C. kelleri*, CASENT0317629).

**Figure 16:**
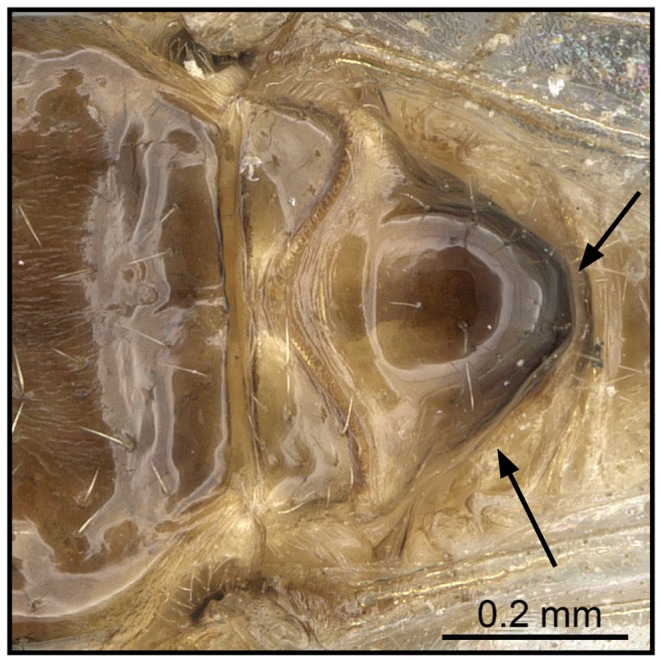
Species-key to the *C. kelleri*-group. Scutellum in dorsal view tapering greatly from anterior to posterior end, dorsoposterior part truncate (*C. kelleri*, CASENT0317629).

**Figure 17:**
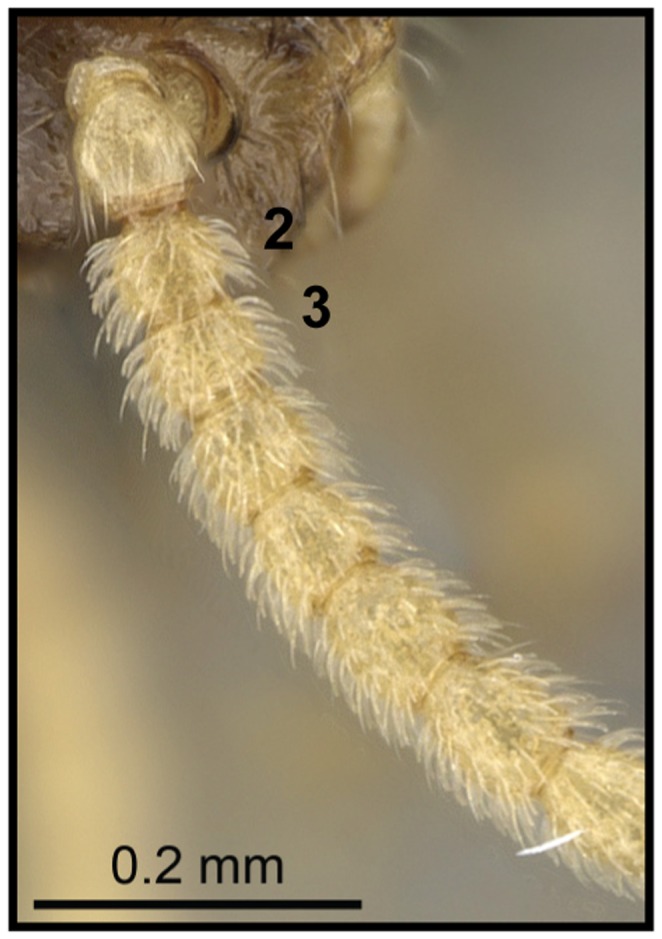
Species-key to the *C. kelleri*-group. Antennae with second and third funicular segment globular, as long as wide (*C. hazolava*, CASENT0317643).

**Figure 18:**
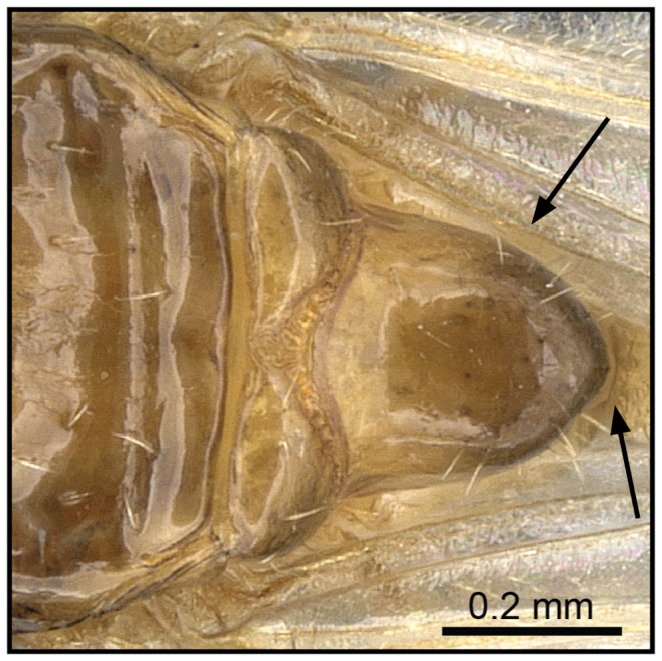
Species-key to the *C. kelleri*-group. Scutellum in dorsal view moderately tapering, dorsoposteriorly pointed (*C. hazolava*, CASENT0317643).

- 1 Antennae with second and third funicular segment longer than wide (Fig. 15); scutellum in dorsal view tapering greatly from anterior to posterior end, dorsoposterior part truncate (Fig. 16).…**2**
  – Antennae with second and third funicular segment globular, as long as wide (Fig. 17); scutellum in dorsal view moderately tapering….**3**
- 2 (1) Body size medium (HW 0.63, WL 1.18)….***C. tavaratra***
  – Body size medium to small (HW 0.47–0.60, WL 0.85–1.19)….***C. kelleri***
- 3 (1) Scutellum and metanotum dorso-posteriorly pointed (Fig. 18)…. ***C. hazolava***
  – Scutellum and metanotum dorso-posteriorly rounded…. ***C. madagascariensis***

### Species Accounts

*Crematogaster kelleri* Forel 1891.

**Figure 19:**
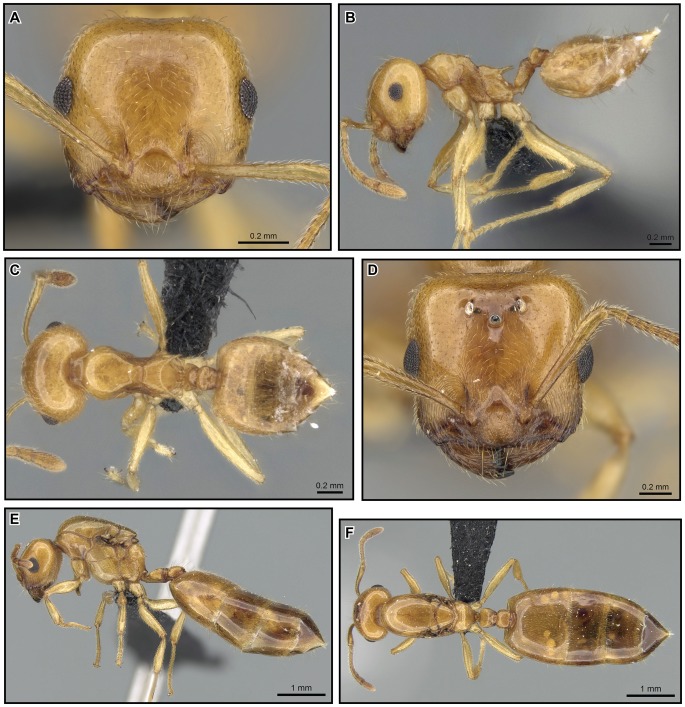
*Crematogaster kelleri*, worker and queen. **A–C** **** worker (CASENT0060043): **A** full face **B** lateral **C** dorsal **D–F** queen (CASENT0124998) **D** full face **E** lateral **F** dorsal.

**Figure 20:**
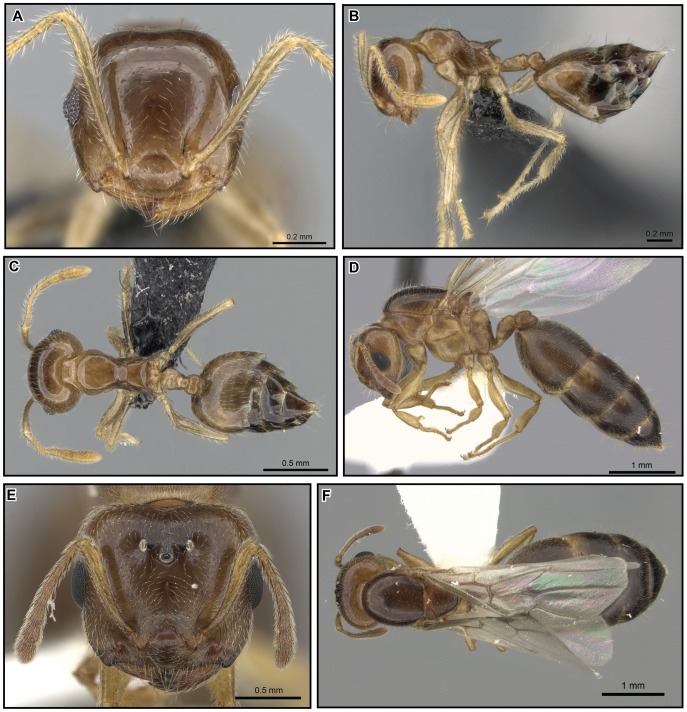
*Crematogaster kelleri*, worker and queen. **A–C** worker (CASENT0107013):**A** full face **B** lateral **C** dorsal **D–F** queen (CASENT0193256) **D** lateral **E** full face **F** dorsal.

**Figure 21:**
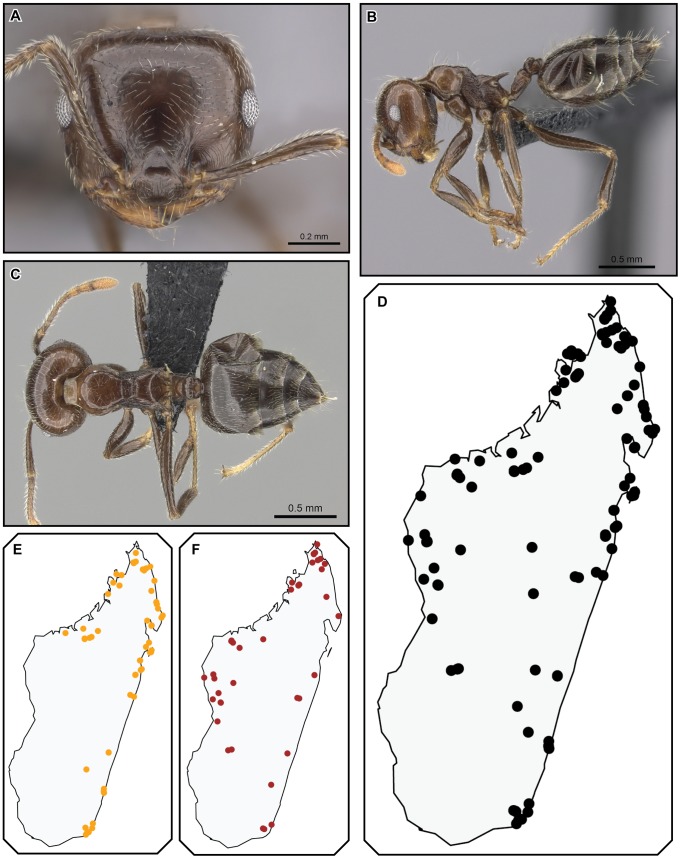
*Crematogaster kelleri*, worker and distribution. **A–C** worker (CASENT0492999): **A** full face **B** lateral **C** dorsal **D–F** distribution: **D** species **E** yellow form **F** brown form.

**Figure 22:**
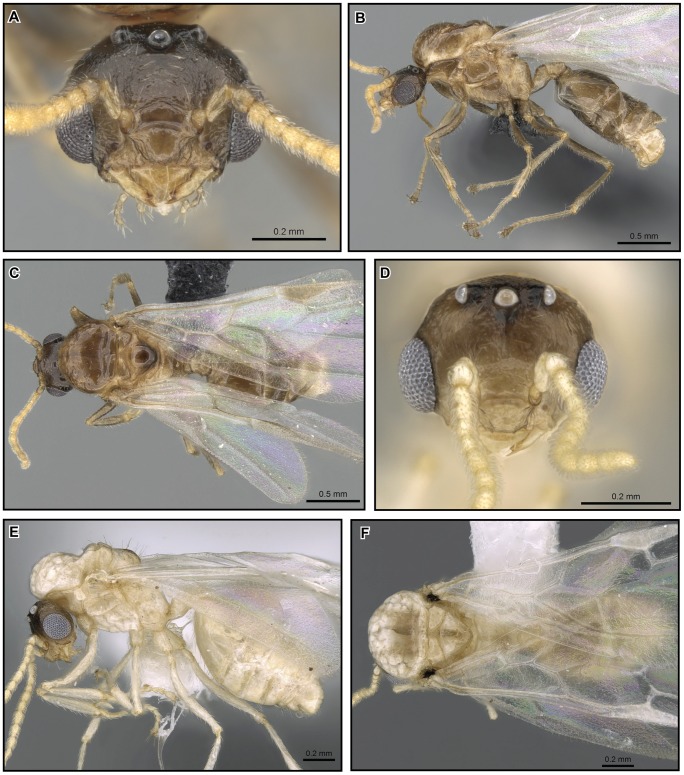
*Crematogaster kelleri*, males. **A–C** CASENT0317629: **A** full face **B** lateral **C** dorsal **D–F** CASENT0193283 **D** full face **E** lateral **F** dorsal.

Figures 19, 20, 21, 22.

*Crematogaster kelleri* Forel, 1891: 197 [34], pl. 6, fig. 10. Worker syntypes from MADAGASCAR: Bois sur les bords de l’Ivondrona, près de Tamatave (C. Keller) **[MHNG, examined].** **Lectotype worker,** by present designation: CASENT0101557, image on AntWeb. Combination in *C. (Crematogaster*): Wheeler, W.M. 1922: 1022 [35]; in *C. (Acrocoelia*): Emery, 1922: 147 [36]; in *C. (Crematogaster*): Bolton, 1995: 166 [37].

 = *Crematogaster adrepens* Forel, 1897: 197. Worker syntypes from MADAGASCAR: Nossi-Bé (Voeltzkow) [**MHNG, examined**]. **Lectotype worker** by present designation: CASENT0101769, top specimen of 3 w on one pin, image on AntWeb. Combination in *C. (Crematogaster*): Wheeler, W.M. 1922: 1022 [35]; in *C. (Acrocoelia*): Emery, 1922: 144 [36]; in *C. (Crematogaster*): Bolton, 1995: 166 [37]. **Syn. nov.**

*= Crematogaster gibba* Emery, 1894: 70 [38], fig. (w.) SEYCHELLES IS. [**MSNG**, **examined**] **Lectotype worker** by present designation: CASENT0102056, top specimen of 2 w on one pin, image on AntWeb. Combination in *C. (Crematogaster*): Wheeler, W.M. 1922: 1022 [35]; in *C. (Acrocoelia*): Emery, 1922: 147 [36]; in *C. (Crematogaster*): Bolton, 1995b: 166 [37]. **Syn. nov.**

#### Type material examined (MHNG)

MADAGASCAR: *Toamasina*: Bois sur les bords de l’Ivondrona [ = Ivondro River], près de Tamatave [-18.23333, 49.36667] (C. Keller), CASENT0101557. Note that Forel described syntypes, but only a single specimen from the type locality was found in the MHNG collection. This is the **lectotype worker,** by present designation: CASENT0101557 (image on AntWeb).

For other material examined (BBBC, CASC, MHNG, PSWC, MCZC) refer to Table S2 in the electronic supporting material.

#### Diagnosis

Workers of *Crematogaster kelleri* can best be separated from *C. tavaratra* by the absence of a distinct, complete longitudinal impression on the postpetiole (complete in the latter). *C. hazolava* and *C. hafahafa* workers can be distinguished from *C. madagascariensis* by the position of the propodeal spiracle, which is not confluent with the spine base. Queens of *C. kelleri* are recognizable from *C. hazolava* queens by their metanotum, which projects below the scutellum, and from *C. tavaratra* queens by the shorter spines (SPI 0.00–0.07; compared to *C. tavaratra* with SPI 0.09–0.10).

#### Worker measurements (n = 44)

HW 0.60–1.16; HL 0.57–0.99; EL 0.12–0.23; SL 0.57–0.84; WL 0.65–1.12; SPL 0.07–0.22; PTH 0.13–0.21; PTL 0.16–0.27; PTW 0.19–0.33; PPL 0.11–0.20; PPW 0.16–0.27; LHT 0.52–0.93; CI 1.04–1.18; OI 0.20–0.25; SI 0.81–1.00; SPI 0.11–0.23; PTHI 0.58–0.86; PTWI 0.94–1.28; PPI 1.34–1.74; LBI 1.14–1.41.

#### Worker description (Figs 19A−C, 20A−C, 21A−C)

Very small to large in size (HW 0.60–1.16, WL 0.65–1.12), but on average small (mean HW 0.81, mean WL 0.82; n = 43); highly morphologically variable, with general characters of the *C. kelleri*-group and the following refinements.

Masticatory margin of mandibles with four, rarely five teeth (in some large specimens); posterior margin of head in full face view laterally subangular, sometimes medially slightly depressed; midline of eyes situated at or slightly above midline of head in full face view.

Promesonotal suture usually absent or indistinct, except in largest workers where mesonotum may be dorsally raised and has a median tubercule; mesonotum with a distinct dorsal and posterior face, and laterally angulate or subangulate; mesonotum with or without posterolateral angular tubercules or denticles, propodeal spines short to medium-sized (SPI 0.11–0.23), spiniform, straight or slightly downcurved, moderately diverging in dorsal view; propodeal spiracle situated below and not confluent with base of spines; postpetiole bilobed, but median impression not fully complete, rather posteriorly deep, anteriorly superficial.

Pilosity highly variable; color yellow or light to dark brown, rarely reddish brown with dark abdominal segments four to seven.

#### Queen measurements (n = 15)

HW 1.15–1.42, HL 1.04–1.29, EL 0.31–0.39, SL 0.71–0.88, MSNW 0.86–1.13, MSNL 0.93–1.21, WL 1.70–2.08, SPL 0.00–0.12, PTH 0.27–0.35, PTL 0.33–0.44, PTW 0.38–0.47, PPL 0.28–0.34, PPW 0.35–0.47, LHT 0.82–1.04, CI 1.05–1.15, OI 0.27–0.32, SI 0.65–0.74, MSNI 0.75–0.98, SPI 0.00–0.07, PTHI 0.66–0.92, PTWI 0.97–1.37, PPI 1.15–1.64, LBI 2.01–2.32.

#### Queen description (Figs 19D−F, 20D−F)

Small (HW 1.15–1.42, WL 1.70–2.08), with characters of the *C. kelleri*-group and the following refinements.

Masticatory margin of mandibles with five teeth; antennal scapes usually not reaching head margin (SI 0.65–0.74); eyes situated at or slightly below midline of head in full face view.

Metanotum projecting from below scutellum in lateral and dorsal view; propodeal spines absent to short (SPI 0.00–0.07), spiniform or dentiform.

#### Male measurements (n = 11)

HW 0.47–0.60, HL 0.39–0.48, EL 0.18–0.33, SL 0.07–0.11, MSNW 0.45–0.72, MSNL 0.42–0.69, WL 0.85–1.19, SPL 0.00, PTH 0.13–0.18, PTL 0.13–0.21, PTW 0.14–0.23, PPL 0.11–0.17, PPW 0.17–0.24, LHT 0.55–0.66, CI 1.17–1.33, OI 0.40–0.68, SI 0.19–0.26, MSNI 0.84–1.39, SPI 0.00, PTHI 0.77–1.11, PTWI 0.84–1.30, PPI 1.16–1.66, LBI 1.63–1.90.

#### Male description (Fig. 22)

Small to medium body size (HW 0.47–0.60, WL 0.85–1.19), with characters of the *C. kelleri*-group and the following refinements.

Masticatory margin of mandibles with two to three teeth; antennae with second and third funicular segment not globular, longer than wide, sixth to eleventh funicular segment distinctly wider than second to fifth segment; occipital carinae distinct, sometimes forming a thin flange projecting backwards.

Scutellum in dorsal view distinctly tapering from anterior to posterior end, dorsoposterior portion truncate; metanotum projecting at least slightly below scutellum in dorsal and lateral view; dorsal face of propodeum very short.

Head sculpture rugulose; scutellum mostly shiny. Color pale yellow to brown, with head darker.

#### Variation

As with many widespread species within the genus *Crematogaster*, *C. kelleri* is morphologically highly variable. We attempted to illustrate this variation with Figures 19A−C, 20A−C and 21A−C. Character states that vary continuously between colonies and to a lesser extent also within colonies are promesonotal structure, color, pilosity and body size. The variable promesonotal structure (i.e. whether the mesonotum is elevated over the pronotum or not) and body size in this species are most likely to cause confusion in specimens not collected from nest series. Fortunately, for our study, recent colony series collections were available in sufficient quantity to estimate variation in this species. Few of these colonies, however, provided queens and even fewer provided males. We found males overall quite variable in size and promesonotal structure, and queens usually variable in size (correlated with worker size).

Color variation in *C. kelleri* appears to be gradual in what is subsequently referred to as the “brown form.” This form can be colored from very pale brown to almost black; the pale coloration is seen mostly in small specimens, whereas darker coloration is most commonly in larger individuals. Color variation in this brown form is also high within colonies, i.e. one colony often has both pale colored, small workers and darker, larger workers. A second color form, the “yellow form”, is distinct from and not part of the variation within the brown form. This yellow color does not appear to be variable within colonies, and colonies with yellow colored workers also have queens of the same coloration. The color of males is unknown. Aside from color, no other character states separate these yellow and brown forms. The lectotype worker of *C. kelleri* represents the yellow form, whereas the syntypes of *C. adrepens* belong to the brown form.

#### Distribution and biology

*Crematogaster kelleri* is widely distributed throughout humid forests and dry forests in Madagascar (Fig. 21D), and has been collected at elevations ranging from sea level up to 2000 m. This species is much more common in lower elevation rainforests and littoral forests than in dry forest or at higher elevations. We therefore surmise that low- to mid-elevation humid forests provide optimal habitat for this species. The yellow form of *C. kelleri* occurs pre-dominantly in regions closer to the coast and at lower elevations and is often the only form found in littoral forests. There is a notable exception to this with an occurrence record at 2000 m in the Andringitra massif. However, since this record does not refer to a colony sample, we consider cross-contamination of samples or mislabeling as a possible explanation. The yellow form is entirely absent from west coast localities (Fig. 21E). The brown form of *C. kelleri* occurs throughout the distribution range in no particular pattern (Fig. 21F). It should be emphasized that both forms can occur in sympatry (e.g. R.N.I. Betampona), but one form usually seems to be more abundant than the other. Furthermore, the species can be found in sympatry with all other species within the *C. kelleri*-group, as well as *C. tsisitsilo*.

*Crematogaster kelleri* typically nests arboreally; collections of colonies are most often made from dead twigs or branches and from nests under bark or canopy moss mats. On occasion, this species has also been found on the ground under stones or in rotten logs. Colonies found under bark or canopy moss appeared to be large and spread across entire trees, while branch-nesters may well have a polydomous nest structure, occupying different parts of one tree.

#### Comments

We found no morphological characters to distinguish the type specimens of *Crematogaster gibba* from *C. kelleri* and therefore synonymize the former under the latter. This conspecificity is peculiar since *C. gibba* was described from the Seychelles, but *C. kelleri* is currently not recorded from these islands. Fisher et al. recently conducted extensive sampling on the Seychelles, including at the type locality Praslin, but did not re-collect the species. In Madagascar, *Crematogaster kelleri* is primarily found in the lowland rainforests. This habitat type is virtually destroyed on the Seychelles, and it is therefore possible that the species is already extinct on these islands.

*Crematogaster madagascariensis* André 1887.

Figure 23, 24.

**Figure 23:**
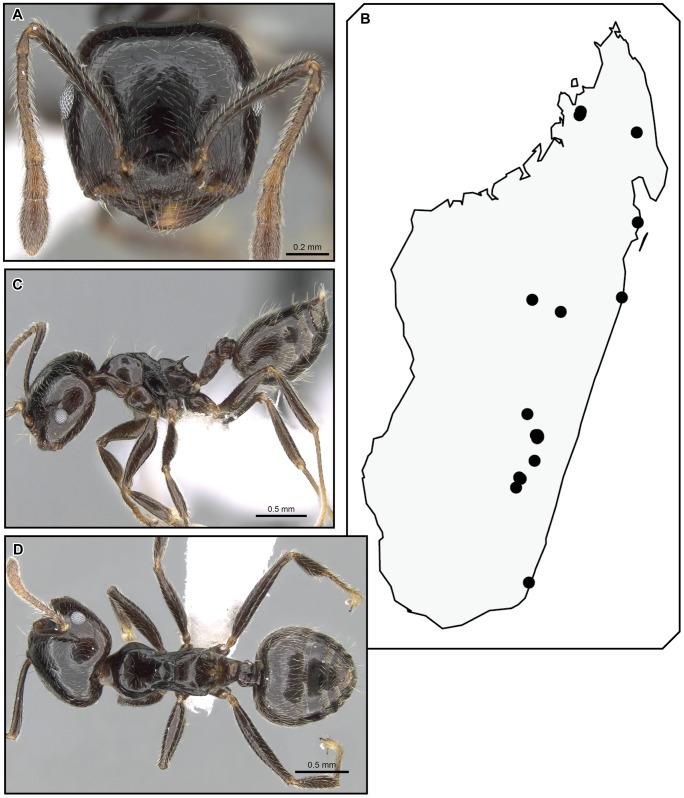
*Crematogaster madagascariensis*, worker and distribution. **A, C, D** worker (CASENT0193446): **A** full face **C** lateral **D** dorsal; **B** species distribution.

**Figure 24:**
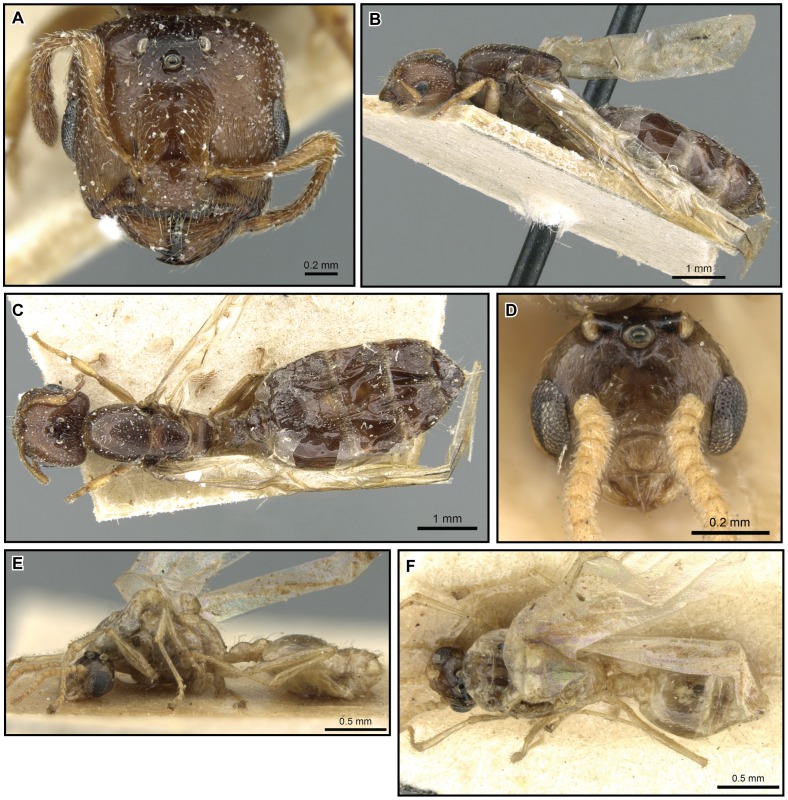
*Crematogaster madagascariensis*, queen and male. **A, B, C** queen (CASENT0906629): **A** full face **B** lateral **C** dorsal; **D, E, F** (CASENT0906631): **D** full face **E** lateral **F** dorsal.

*Crematogaster madagascariensis* André 1887: 297 [39]. Worker, queen and male syntypes from MADAGASCAR: Tamatave (unknown collector) [**ZMHB examined** (MNHN unclear)]. **Lectotype worker** by present designation: CASENT0906628, image on AntWeb. Combination in *C. (Crematogaster*): Wheeler, W.M. 1922: 1023 [35]; in *C. (Acrocoelia*): Emery, 1922: 147 [36]; in *C. (Crematogaster*): Bolton, 1995: 166 [37].

#### Type material

At the MNHN, where the André collection is housed, no specimens from the type locality Tamatave were found. Instead we examined the following (non-type) material from the André collection at MNHN: Madagascar (unknown collector), 5 workers on one pin, middle specimen (CASENT0101409) imaged on AntWeb. However, we examined material from ZMHB with the following type locality information: Madagascar: Tamatave (Friedrichs), 1 w, 1 aQ, 1 m. Given the distribution of other André type material across museums in Europe, it is highly likely that these specimens belong to the syntype series (P.S.Ward, pers. comm.) and we therefore consider this material eligible for lectotype designation (see above).

For **other material examined** (BBBC, CASC, MHNG, ZMHB, MCZC) refer to Table S2 in the electronic supporting material.

#### Diagnosis

Within the *C. kelleri*-group, workers of *C. madagascariensis* can be distinguished from *C. kelleri* and *C. tavaratra* by the position of the propodeal spiracle, which is situated confluent with the base of the propodeal spines in *C. madagascariensis*, but below the spine base in the two latter species. From the two remaining species, workers are most easily separated by body size and coloration: *C. madagascariensis* workers are usually larger (HW 0.86–1.11, WL 0.92–1.16) than *C. hazolava* (HW 0.66–0.89, WL 0.70–0.97) and *C. hafahafa* (HW 0.68–0.72, WL 0.73–0.74) workers, are brown-colored (yellow in *C. hafahafa* and some *C. hazolava* populations) and lack the median postpetiolar stripe (of brown-colored *C. hazolava* workers, see Fig. 6).

#### Worker measurements (n = 22)

HW 0.86–1.11; HL 0.78–0.99; EL 0.15–0.23; SL 0.67–0.86; WL 0.92–1.16; SPL 0.08–0.18; PTH 0.15–0.22; PTL 0.22–0.31; PTW 0.24–0.37; PPL 0.15–0.20; PPW 0.22–0.30; LHT 0.68–0.97; CI 1.07–1.18; OI 0.18–0.27; SI 0.79–0.96; SPI 0.08–0.15; PTHI 0.56–0.83; PTWI 0.93–1.31; PPI 1.33–1.71; LBI 1.04–1.50.

#### Worker description (Fig. 23A−C)

Small to large species (HW 0.86–1.11, WL 0.92–1.16), with characters of the *C. kelleri*-group, in addition to the following.

Masticatory margin of mandibles with four teeth; posterior margin of head in full face view laterally subangular, sometimes medially slightly depressed; midline of eyes situated slightly above midline of head in full face view.

Lateral borders of mesonotum angulate, and mesonotum with posterolateral angular tubercules; posterior face of mesonotum sloping more gently into metanotal groove; propodeal spines short (SPI 0.07–0.15), spiniform, straight or downcurved, moderately diverging in dorsal view; propodeal spiracle confluent with base of propodeal spines; median postpetiolar impression usually deep, clearly bisecting postpetiole into two lobes. Color light to dark brown.

#### Queen measurements (n = 1)

HW 1.39, HL 1.26, EL 0.38, SL 0.82, MSNW 1.01, MSNL 1.26, WL 2.21, SPL 0.06, PTH 0.39, PTL 0.43, PTW 0.51, PPL 0.39, PPW 0.55, LHT n.a., CI 1.10, OI 0.30, SI 0.65, MSNI 0.80, SPI 0.03, PTHI 0.91, PTWI 1.18, PPI 1.40, LBI n.a.

#### Queen description (Figs 24A–C)

Medium (HW 1.39, WL 2.21), with characters of the *C. kelleri*-group and the following refinements.

Masticatory margin of mandibles with five teeth; antennal scapes usually not reaching head margin (SI 0.65); eyes situated at midline of head in full face view.

Metanotum not projecting from below scutellum in lateral and dorsal view; propodeal spines short (SPI 0.06), dentiform.

#### Male measurements (n = 3)

HW 0.53–0.59, HL 0.40–0.50, EL 0.20–0.22, SL 0.09, MSNW 0.56–0.71, MSNL 0.65–0.74, WL 1.02–1.18, SPL 0.00, PTH 0.16, PTL 0.19–0.25, PTW 0.18–0.19, PPL 0.11–0.14, PPW 0.19–0.25, LHT 0.62–0.71, CI 1.19–1.31, OI 0.44–0.50, SI 0.19–0.22, MSNI 0.86–0.96, SPI 0.00, PTHI 0.63–0.83, PTWI 0.63–0.75, PPI 1.44–2.00, LBI 1.65–1.90.

#### Male description (Fig. 24D–F)

Small to medium body size (HW 0.53–0.59, WL 1.02–1.18), with characters of the *C. kelleri*-group and the following refinements.

Masticatory margin of mandibles with two teeth; antennae with second and third funicular segment globular; occipital carinae indistinct.

Scutellum in dorsal view little tapering from anterior to posterior end; metanotum not projecting below scutellum in dorsal and lateral view; dorsal face of propodeum absent.

Head sculpture rugulose; scutellum mostly shiny. Color light brown, with head darker.

**Distribution and biology.** *Crematogaster madagascariensis* is distributed primarily in mid-elevation to montane rainforest in the central highlands region of Madagascar (Fig. 23B). Curious exceptions are its presence at lower elevations in R.S. Manongarivo and P.N. Marojejy in the north, and in P.N. Mananara-Nord and Forêt Ivohibe on the east coast. The type locality for this species is Tamatave on the east coast of Madagascar; however, the species has not been collected recently from this area. This suggests that this species could have been historically more common in lower elevation forests in Madagascar, but habitat destruction may have resulted in a more limited present-day distribution. *Crematogaster madagascariensis* occurs in sympatry with *C. kelleri* and *C. hazolava*. Very few colony collections of this species have been made and thus not much is known about its biology. We presume an arboreal lifestyle.

#### Comments

A fruitless effort has been made to locate the syntype material of this species in the collection of E. André at the MNHN [e-mail communication with Mlle Touret-Alby, 31.iii.2011]; no material from the type locality [Ivondro River, close to Tamatave] was to be found in this collection. Since all other material that was later added to the collection by André himself conforms to the species description, and we found material in the ZMHB collection from the type locality that could belong to the syntype material, we are nonetheless confident in the correct identification of this taxon.

*Crematogaster hafahafa* Blaimer **sp. nov.**

urn:lsid:zoobank.org:act:EF4E3671-50EB-4E2B-9C2F-1F8AE 8C18520.

Fig. 25.

**Figure 25:**
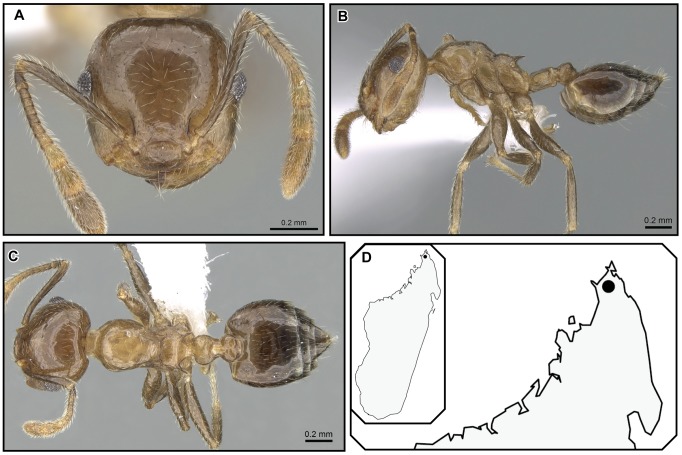
*Crematogaster hafahafa*, worker and distribution. **A–C** worker (CASENT0436524): **A** full face **B** lateral **C** dorsal; **D** species distribution.

#### Type locality

MADAGASCAR: *Antsiranana*: P.N. Montagne d’Ambre: -12.53444, 49.17950, 925 m, montane rainforest.

#### Type specimens

**holotype** worker: pinned, CASENT0436524, BLF02566(18), beating low vegetation, 20.−26.i.2001, B. L. Fisher et al.; original locality label: MADG’R: Prov. Antsiranana: P.N. Montagne Ambre, 3.6 km 235° SW Joffreville, 925 m, 20.−26.i.2001, Fisher et al. BLF2566; deposited at CASC.

#### Other material examined (CASC)

MADAGASCAR: P.N. Montagne d’Ambre, 925 m.

#### Diagnosis

*Crematogaster hafahafa* can be distinguished easily from *C. kelleri* by the shape of the promesonotum, which is rounded in lateral view in *C. hafahafa* (Fig. 25B), in contrast to the characteristic shape (Fig. 19B, 20B, 21B) in *C. kelleri*. *Crematogaster hafahafa* is not known to co-occur with any other species besides *C. kelleri* within the *C. kelleri*-group.

#### Worker measurements (n = 2) [holotype]

HW 0.68–[0.72]; HL 0.63–[0.65]; EL 0.14–[0.15]; SL 0.56; WL 0.73–[0.74]; SPL 0.09; PTH 0.14–[0.15]; PTL [0.17]–0.18; PTW 0.22; PPL 0.13; PPW 0.20; LHT 0.57; CI 1.08–[1.11]; OI 0.22–[0.23]; SI [0.86]–0.89; SPI 0.12; PTHI 0.78– [0.87]; PTWI 1.20–[1.31]; PPI 1.48–[1.49]; LBI 1.29.

#### Worker description (Fig. 25A−C)

Very small species (HW 0.68–0.72, WL 0.73–0.74), with characters of the *C. kelleri*-group, except for the following refinements.

Masticatory margin of mandibles with four teeth; posterior margin of head in full face view subangularly rounded, sometimes medially slightly depressed; midline of eyes situated slightly above midline of head in full face view; eyes distinctly protruding from lateral head margin.

Promesonotum rounded in lateral view; lateral borders of mesonotum subangulate, posterolateral tubercules or denticles absent; posterior face of mesonotum sloping into metanotal groove at 45°; propodeal spines short (SPI 0.12), spiniform, downcurved, moderately diverging in dorsal view; propodeal spiracle situated slightly off-center and lateral with respect to propodeal spines; petiole in dorsal view short and oval, subpetiolar process undeveloped; median postpetiolar impression usually deep, clearly bisecting postpetiole into two lobes.

Pilosity within range as described for *C. kelleri*-group, but on the less abundant end. Color pale yellow, abdominal segments four to seven darker.

#### Distribution and biology

*Crematogaster hafahafa* is only known from two specimens collected in mid-elevation rainforest in the Montagne d’Ambre massif (Fig. 25D), where it occurs in sympatry with *C. kelleri.* Nothing is known about the natural history of this species since the sole collection was made by beating vegetation.

#### Etymology

This species is named “hafahafa”, meaning peculiar or odd in Malagasy, for its odd appearance and rarity. The name is treated as a noun in apposition.

*Crematogaster hazolava* Blaimer **sp. nov.**

urn:lsid:zoobank.org:act:B5FE9290-239F-4CF8-A119-73669AB0D58D.

Figures 26, 27.

**Figure 26:**
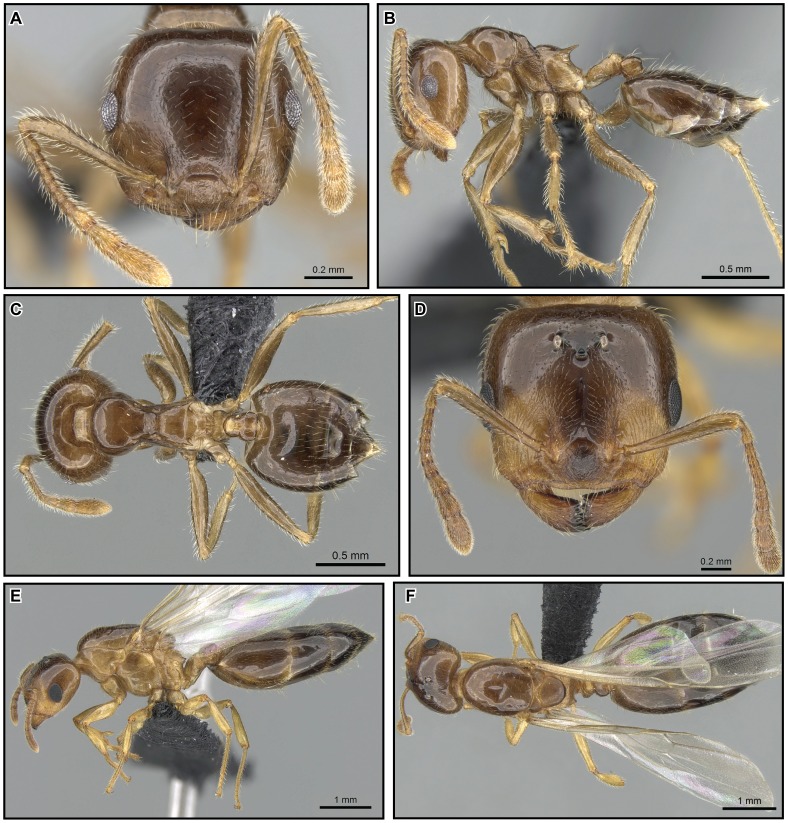
*Crematogaster hazolava,* worker and queen. **A–C** worker (CASENT0317642): **A** full face **B** lateral **C** dorsal **D–F** queen (CASENT0160002) **D** full face **E** lateral **F** dorsal.

**Figure 27:**
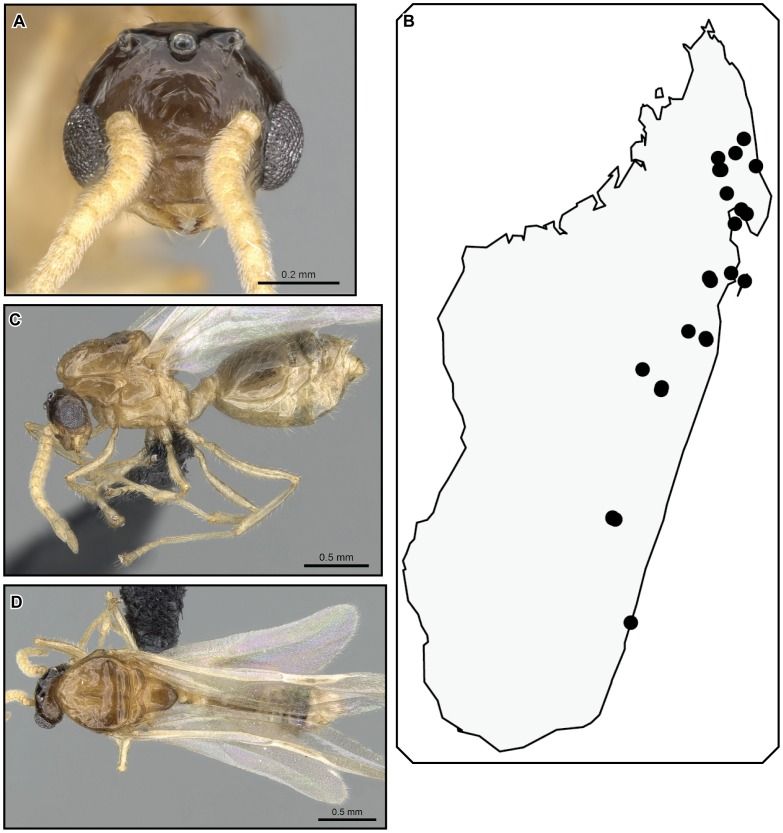
*Crematogaster hazolava*, male and distribution. **A, C, D** male (CASENT0317643): **A** full face **C** lateral **D** dorsal; **B** species distribution.

#### Type locality

MADAGASCAR: *Toamasina*: R.N.I. Betampona: -17.91106, 49.21111, 395−445 m, rainforest.

#### Type specimens

**holotype** worker: pinned, CASENT0317642 (image on AntWeb), B-I-2b-6-GC4, ex dead branch 6 m above ground, 22.vi.2012, E. H. Lokensgard; original locality label: M’dgascar: Prov. Toamasina: R.N.I. Betampona, -17.91106, 49.21111, 395−445 m, 10.vi.−2.vii.12, B. B. Blaimer et al., arboreal coll., B-I-2b-6-GC4; deposited at CASC.

Four **paratype** workers, pinned, same collection and locality data as holotype. #1: CASENT0317679, deposited at SAMC; #2: CASENT0317680, deposited at MHNG. #3: CASENT0317681, deposited at MCZC. #4: CASENT0317682, deposited at UCDC.

For **other material examined** (BBBC, CASC, PSWC) refer to Table S2 in the electronic supporting material.

#### Diagnosis

Within the *C. kelleri*-group, workers of *C. hazolava* can be separated from *C. kelleri* and *C. tavaratra* by the position of the propodeal spiracle, which is situated confluent with the base of the propodeal spines in *C. hazolava*, instead of below the spine base in the two latter species. From *C. madagascariensis* workers are most easily separated by body size and coloration, the former being mostly larger (HW 0.86–1.11, WL 0.92–1.16) than *C. hazolava* (HW 0.66–0.89, WL 0.70–0.97). Brown-colored workers of *C. hazolava* could potentially be confused with *C. madagascariensis*, but these can be distinguished by the median postpetiolar stripe. *Crematogaster hazolava* does not co-occur with *C. hafahafa*, but in any case workers could potentially be distinguished by larger size and the presence of the median postpetiolar stripe. Queens of *C. hazolava* can be differentiated from *C. kelleri* and *C. tavaratra* queens easily since the metanotum is entirely covered by the scutellum in the former, but projects from below the metanotum in the latter two species. From *C. madagascariensis* queens of *C. hazolava* can be distinguished by the presence of the median longitudinal stripe on the postpetiole.

#### Worker measurements (n = 31) [holotype]

HW 0.66–0.89 [0.84]; HL 0.63–0.84 [0.79]; EL 0.14–0.18 [0.16]; SL 0.58–0.74 [0.69]; WL 0.70–0.97 [0.90]; SPL 0.07–0.12 [0.12]; PTH 0.13–0.17 [0.17]; PTL 0.19–0.26 [0.24]; PTW 0.21–0.28 [0.22]; PPL 0.11–0.16 [0.16]; PPW 0.17–0.25 [0.22]; LHT 0.54–0.72 [0.66]; CI 1.04–1.13 [1.06]; OI 0.19–0.23 [0.20]; SI 0.82–0.95 [0.88]; SPI 0.07–0.14 [0.13]; PTHI 0.61–0.75 [0.73]; PTWI 0.82–1.08 [0.94]; PPI 1.33–1.68 [1.37]; LBI 1.27–1.58 [1.36].

#### Worker description (Fig. 26A−C)

Very small to small species (HW 0.66–0.89, WL 0.70–0.97), with characters of the *C. kelleri*-group and the following refinements.

Masticatory margin of mandibles with four teeth; posterior margin of head in full face view laterally rounded, often medially depressed; midline of eyes situated at midline of head in full face view.

Lateral borders of mesonotum angulate to carinulate, and mesonotum with small posterolateral denticles; posterior face of mesonotum sloping into metanotal groove at 45°; propodeal spines short (SPI 0.07–0.12), spiniform, straight, moderately diverging in dorsal view; propodeal spiracle confluent with base of propodeal spines; petiole suboval, dorsolateral carinulate; median postpetiolar impression deep, clearly bisecting postpetiole into two lobes, and usually lighter colored, appearing as longitudinal stripe (can be difficult to see in yellow specimens).

Erect pilosity and pubescence highly variable, but usually abundant. Color brown or yellow, abdominal segments five through seven often darker.

#### Queen measurements (n = 3)

HW 1.28–1.37, HL 1.20–1.24, EL 0.32–0.36, SL 0.80–0.88, MSNW 1.01–1.13, MSNL 1.17–1.27, WL 1.99–2.07, SPL 0.04–0.09, PTH 0.28–0.34, PTL 0.37–0.38, PTW 0.39–0.48, PPL 0.29–0.34, PPW 0.37–0.58, LHT 0.92–0.94, CI 1.07–1.09, OI 0.29, SI 0.65–0.67, MSNI 0.83–0.96, SPI 0.02–0.04, PTHI 0.84–0.91, PTWI 1.04–1.30, PPI 1.06–1.50, LBI 2.16–2.25.

#### Queen description (Fig. 26D−F)

Small (HW 1.28–1.37, WL 1.99–2.07), with characters of the *C. kelleri*-group in addition to the following.

Masticatory margin of mandibles with five to six teeth; antennal scapes short, barely reaching beyond level of ocelli; eyes situated distinctly below midline of head in full face view.

Mesonotum covered entirely by scutellum in dorsal view; propodeal spines very short spines or denticles (SPI 0.02–0.04).

#### Male measurements (n = 2)

HW 0.59–0.65, HL 0.49–0.51, EL 0.24–0.26, SL 0.09, MSNW 0.67–0.76, MSNL 0.66–0.76, WL 1.15–1.24, SPL 0.00, PTH 0.16–0.17, PTL 0.21–0.23, PTW 0.21–0.23, PPL 0.10–0.13, PPW 0.20–0.26, LHT 0.62–0.66, CI 1.20–1.27, OI 0.50–0.51, SI 0.18–0.19, MSNI 1.00–1.01, SPI 0.00, PTHI 0.85–0.94, PTWI 1.11–1.18, PPI 2.05–2.08, LBI 1.86–1.88.

#### Male description (Fig. 27A,C,D)

Medium body size (HW 0.59–0.65, 1.15–1.24). Masticatory margin of mandibles with three teeth; antennae with second and third funicular segment globular, as long as wide, and all funicular segments evenly wide; in full face view ocellar triangle situated at posterior head margin and slightly elevated with respect to rest of face; occipital carinae very distinct, but not forming a thin flange that projects backwards.

Mesoscutum in dorsal view round; scutellum in dorsal view laterally pinched, but evenly so and not tapering from anterior to posterior end, dorsoposterior portion pointed; mesonotum projecting posteriorly as a small point, but nonetheless entirely covered in dorsal view by scutellum; dorsal face of propodeum absent.

Head sculpture shiny throughout; face with a paired row of short erect setae on frons; mesoscutum with scattered short erect pilosity. Color as in worker and queen, head darker.

#### Distribution and biology

*Crematogaster hazolava* is moderately common in humid forests of eastern Madagascar (Fig. 27B), with a few disjunct occurrences in the central highland region (P.N. Ranomafana, Ambatovy, Analamay). The species seems predominantly adapted to littoral, low and mid elevation forests, but has been collected from a wider altitudinal range (20–1300 m). It is found in sympatry with *C. madagascariensis* and *C. kelleri*. Curiously, this species also has a yellow, less common form that partly co-occurs with the yellow form of *C. kelleri*. Many colonies of *C. hazolava* have been collected nesting in dead twigs or branches, and a few have been found under bark. This species appears to nest exclusively arboreally. When found together with *C. kelleri*, this species occurred only in low abundance: during a canopy study at R.N.I Betampona that sampled 48 trees, only seven colonies of *C. hazolava* (in comparison to 18 colonies of *C. kelleri*) were obtained.

#### Etymology

“Hazolava” means “tall tree” in Malagasy, after the species’ association with rainforest habitats and preference for arboreal nesting habits. The name should be treated as a noun in apposition.

*Crematogaster tavaratra* Blaimer **sp. nov.**

urn:lsid:zoobank.org:act:29073F39-9632-42F3-80D5-1400ACA ECDF6.

Figures 28, 29.

**Figure 28:**
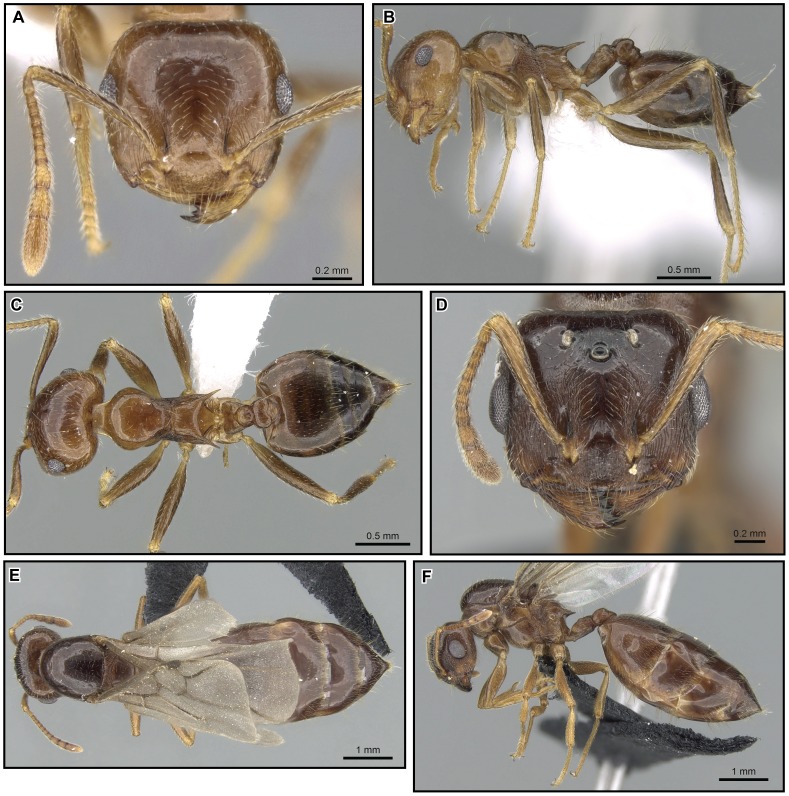
*Crematogaster tavaratra*, worker and queen. **A–C** worker (CASENT0436456):**A** full face **B** lateral **C** dorsal **D–F** queen (CASENT0317694): **D** full face **E** dorsal **F** lateral.

**Figure 29:**
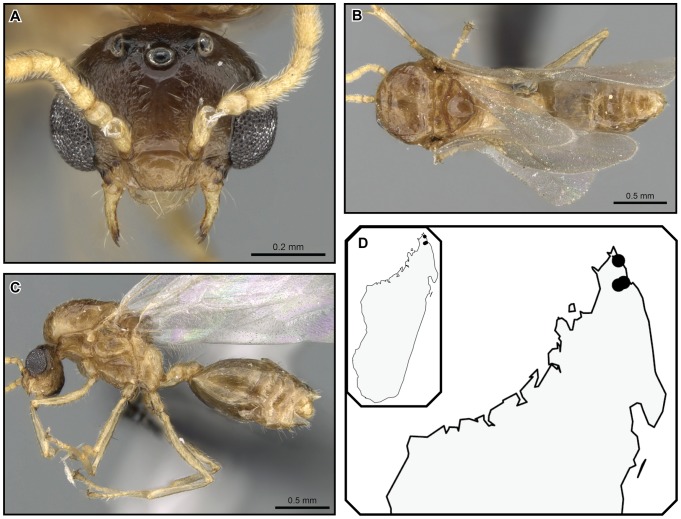
*Crematogaster tavaratra*, male and distribution. **A–C** male (CASENT0110524): **A** full face **B** lateral **C** dorsal; **D** species distribution.

#### Type locality

MADAGASCAR: *Antsiranana*: Forêt Orangea: -12.25889, 49.37467, 90 m, littoral forest.

#### Type specimens

**holotype** worker: pinned, CASENT0436456, BLF03207, ex dead twig above ground [imaged on Antweb]; original locality label: MADG’R: Prov. Antsiranana, Forêt Orangea, 3.6 km 128° SE Remena 90 m, 12°16′S 49°22′E, 22–28.ii.2001, Fisher et al., BLF3207; deposited at CASC.

Four **paratype** workers, pinned, same collection and locality data as holotype. #1: CASENT0317674, deposited at SAMC; #2: CASENT0317675, deposited at MHNG. #3: CASENT0317676, deposited at MCZC. #4: CASENT0317677, deposited at UCDC.

For **other material examined** (CASC) refer to Table S2 in the electronic supporting material.

#### Diagnosis

Workers of *Crematogaster tavaratra* can be separated from *C. kelleri* by the distinct longitudinal impression on the postpetiole (incomplete in the latter). The species does not co-occur with any of the other species (*C. madagascariensis*, *C. hazolava*, *C. hafahafa*) in the *C. kelleri*-group, but could not otherwise be confused given the distinctly longer propodeal spines and the position of the propodeal spiracle below and not confluent with the spine base. Queens of *C. tavaratra* can be separated easily from other (known) queens of the *C. kelleri*-group by their distinct longer spines (SPI 0.09–0.10; compared to *C. kelleri* and *C. hazolava* SPI 0.00–0.07).

#### Worker measurements (n = 13) [holotype]

HW 0.83–1.03 [0.96]; HL 0.73–0.93 [0.86]; EL 0.15–0.22 [0.20]; SL 0.70–0.83 [0.78]; WL 0.90–1.12 [1.07]; SPL 0.17–0.27 [0.23]; PTH 0.17–0.21 [0.21]; PTL 0.25–0.29 [0.28]; PTW 0.25–0.30 [0.29]; PPL 0.17–0.20 [0.17]; PPW 0.23–0.29 [0.29]; LHT 0.71–0.91[0.86]; CI 1.11–1.16 [1.11]; OI 0.19–0.25 [0.23]; SI 0.86–0.99 [0.90]; SPI 0.17–0.24 [0.21]; PTHI 0.60–0.85 [0.74]; PTWI 0.91–1.19 [1.02]; PPI 1.28–1.68 [1.67]; LBI 1.18–1.27 [1.24].

#### Worker description (Fig. 28A−C)

Small to medium-sized species (HW 0.83–1.03, WL 0.90–1.12), with general characters of the *C. kelleri*-group and the following refinements.

Masticatory margin of mandibles with five teeth; posterior margin of head in full face view laterally subangular, often medially slightly depressed; midline of eyes situated above midline of head in full face view.

Lateral borders of mesonotum subangulate, posterolateral denticles absent; promesonotum planar, posterior face of mesonotum sloping into metanotal groove at ca. 45° angle; propodeal spines medium-sized (SPI 0.17–0.24), spiniform, straight, moderately diverging in dorsal view; propodeal spiracle situated distinctly below and not confluent with base of propodeal spines; petiole suboval to moderately flared, dorsolateral not carinulate, but with minute posterolateral denticles bearing paired erect setae; subpetiolar process developed into an acute tooth; median postpetiolar impression deep, clearly bisecting postpetiole into two lobes.

Face with 8–14 erect, long, flexuous setae; promesonotum usually with eight to ten long, flexuous setae: four to six pronotal setae, and two lateral setae each on dorsal and posterior face of mesonotum; abdominal tergites and sternites four through seven with abundant long erect pilosity, and short appressed to decumbent pubescence throughout. Color either yellow or dirty-orange with abdominal segments four through seven brown or black colored, or uniformly brown.

#### Queen measurements (n = 5)

HW 1.38–1.45, HL 1.18–1.25, EL 0.36–0.40, SL 0.87–0.95, MSNW 1.13–1.18, MSNL 1.24–1.30, WL 2.26–2.34, SPL 0.19–0.24, PTH 0.32–0.38, PTL 0.42–0.47, PTW 0.49–0.55, PPL 0.31–0.35, PPW 0.39–0.52, LHT 1.07–1.13, CI 1.11–1.20, OI 0.30–0.32, SI 0.72–0.77, MSNI 0.87–0.96, SPI 0.09–0.10, PTHI 0.67–0.86, PTWI 1.03–1.31, PPI 1.13–1.61, LBI 2.06–2.16.

#### Queen description (Fig. 28D−F)

Medium size (HW 1.38–1.45, WL 2.26–2.34), with characters of the *C. kelleri*-group in addition to the following.

Masticatory margin of mandibles with five teeth; antennal scapes reaching or slightly surpassing head margin; eyes situated at midline of head in full face view.

Metanotum projecting from below scutellum in dorsal and lateral view; propodeal spines longer and spiniform (SPI 0.09–0.10).

#### Male measurements (n = 1)

HW 0.63, HL 0.46, EL 0.26, SL 0.11, MSNW 0.77, MSNL 0.69, WL 1.18, SPL 0.00,PTH 0.19, PTL 0.21, PTW 0.21, PPL 0.16, PPW 0.21, LHT 0.65, CI 1.35, OI 0.56, SI 0.23, MSNI 1.11, SPI 0.00, PTHI 0.89, PTWI 1.00, PPI 1.33, LBI 1.82.

#### Male description (Fig. 29A−C)

Medium body size (HW 0.63, WL 1.18). Masticatory margin of mandibles with two to three teeth (in the single specimen examined right mandible with three teeth, left mandible with two teeth); antennae with second and third funicular segments longer than wide, and sixth to eleventh funicular segments distinctly wider than second to fifth segments; in full face view ocellar triangle situated below posterior head margin and not elevated with respect to rest of face; occipital carinae very distinct, forming a thin flange that projects backwards.

Mesoscutum in dorsal view oval; scutellum in dorsal view laterally pinched, tapering greatly from anterior to posterior end, dorsoposterior part truncate; mesonotum short, with median carinae; dorsal face of propodeum very short; propodeal spines absent.

Head sculpture rugulose around eyes and ocelli; face with two short erect setae and very sparse suberect pubescence; mesoscutum with scattered short erect pilosity. Color as in worker and queen, with head darker.

#### Distribution and biology

*Crematogaster tavaratra* is known only from two dry deciduous forests in the very far north of Madagascar, Réserve Analamerana and Forêt d’Orangea, at elevations between 60−225 m (Fig. 29D). This species co-occurs with *C. kelleri* at the first locality. Colony collections of *C. tavaratra* were made from dead branches and suggest that this species nests arboreally.

#### Etymology

This species is named after its extreme restriction to northern Madagascar: “tavaratra” is Malagasy for “those from the north”. This name should be treated as noun in apposition.

*Crematogaster tsisitsilo* Blaimer **sp. nov.**

urn:lsid:zoobank.org:act:32749542-A8A4-42D7-A355-35300B0 15E32.

Figures 30, 31.

**Figure 30:**
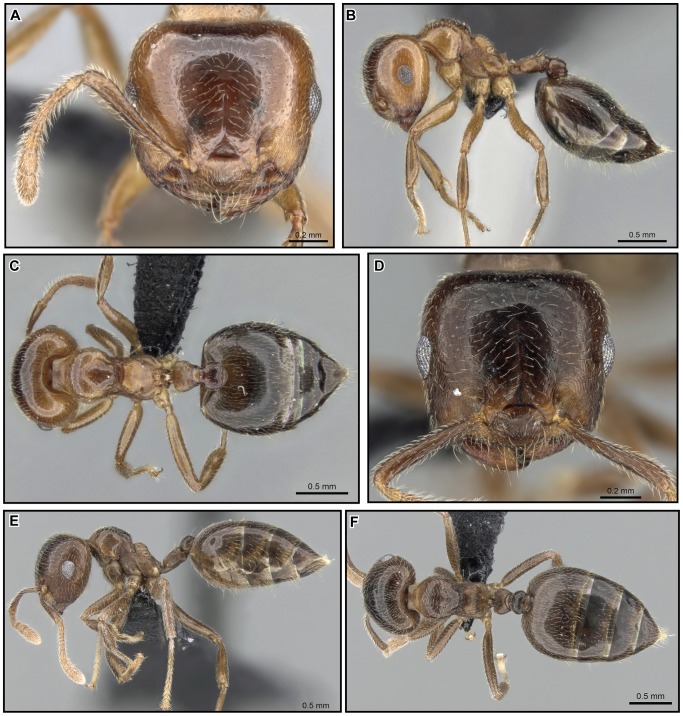
*Crematogaster tsisitsilo*, workers. **A–C** CASENT0120279: **A** full face **B** lateral **C** dorsal **D–F** CASENT0317686: **D** full face **E** lateral **F** dorsal.

**Figure 31:**
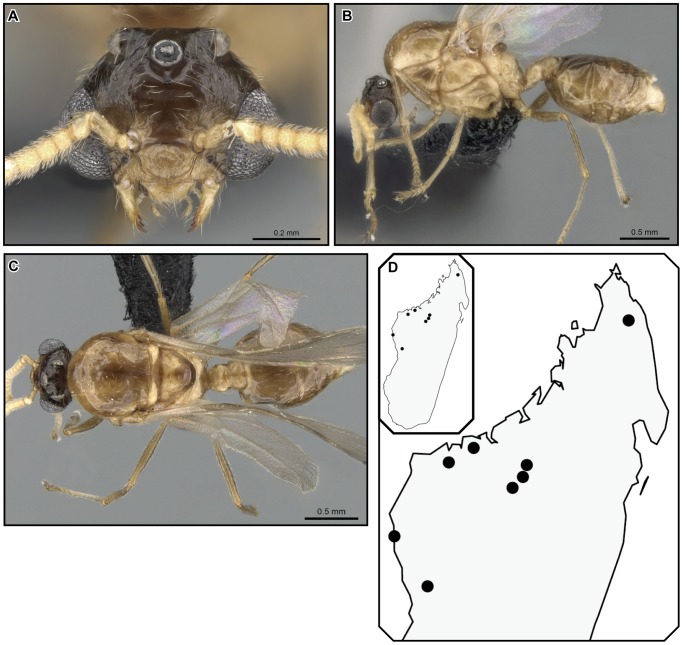
*Crematogaster tsisitsilo*, male and distribution. **A–C** male (CASENT0317695): **A** full face **B** lateral **C** dorsal; **D** species distribution.

#### Type locality

MADAGASCAR: *Mahajanga*: Ambondromamy: -16.43750, 47.15750, 64 m, urban/garden.

#### Type specimens

**holotype** worker: pinned, CASENT0317686, BLF16530, on low vegetation [imaged on Antweb]; original locality label: MADG’R: Majunga: Ambondromamy:, 64 m, 07.ii.2007, 16°26.25′S, 47°09.45′E, urban/garden, Fisher et al., BLF16530; deposited at CASC.

Four **paratype** workers, pinned, same collection and locality data as holotype. #1: CASENT0317684, deposited at SAMC; #2: CASENT0317685, deposited at MHNG. #3: CASENT0317687, deposited at MCZC. #4: CASENT0317688, deposited at UCDC.

For **other material examined** (CASC, MCZC) refer to Table S2 in the electronic supporting material.

#### Diagnosis

*Crematogaster tsisitsilo* is uniquely recognizable from all other *Crematogaster* species in Madagascar by the lack of propodeal spines and the absence of a median impression on the postpetiole.

#### Worker measurements (n = 20)

HW 0.66−1.03 [0.97]; HL 0.62−0.91 [0.89]; EL 0.13−0.23 [0.21]; SL 0.46−0.67 [0.66]; WL 0.62−0.98 [0.95]; SPL 0.00; PTH 0.12−0.21 [0.21]; PTL 0.18−0.31 [0.28]; PTW 0.21−0.32 [0.32]; PPL 0.12−0.20 [0.17]; PPW 0.18−0.30 [0.30]; LHT 0.42−0.73 [0.69]; CI 1.07−1.15 [1.09]; OI 0.21−0.27 [0.23]; SI 0.73−0.82 [0.75]; SPI 0.00; PTHI 0.65−0.83 [0.76]; PTWI 0.99−1.29 [1.15]; PPI 1.29−1.81 [1.81]; LBI 1.30−1.63 [1.37].

#### Worker description (Fig. 30A−F)

Very small to medium size (HW 0.66−1.03, WL 0.62−0.98).

Masticatory margin of mandibles with four teeth; head shape quadrate, mostly as long as wide (CI 1.07–1.15); posterior margin of head in full face view laterally rounded, subangular or angular; occipital carinae indistinct; antennal scapes not, or just reaching head margin; midline of eyes situated at midline of head in full face view; eyes fairly large (OI 0.21–0.27) and moderately protruding.

Pronotum laterally subangular; promesonotal suture distinct or indistinct; mesonotum to varying degree raised over pronotum, and a minute median tubercule is present; mesonotum usually with a rounded dorsal and a short posterior face; mesonotum rounded dorsolaterally; in lateral view outline of promesonotum appears as one convex hump; posterior face of mesonotum slopes steeply into metanotal groove; metanotal groove fairly deep, much constricted and bordered by lateral carinae; propodeal spines absent; propodeal spiracles large; dorsal face of propodeum very short and convex, distinctly set off from posterior face, laterally expanded to about twice the width of metanotal groove; posterior face of propodeum convex and steeply sloping; petiole in dorsal view oval, dorsolaterally rounded and without posterolateral tubercules or denticles; subpetiolar process absent; postpetiole wider than long, rounded, not distinctly bilobed, with at most a faint median impression posteriorly; subpostpetiolar process absent.

Sculpture reduced overall; head shiny to aciculate; mesosoma dorsally shiny and aciculate; propleuron aciculate, meso- and metapleuron mostly reticulate; dorsal face of propodeum aciculate to reticulate, posterior face shiny; dorsal face of petiole shiny; helcium aciculate; postpetiole dorsally aciculate; lateral and ventral face of petiole and postpetiole feebly reticulate. Face usually with four to eight erect, longer setae, and abundant shorter erect to subdecumbent pubescence; usually two to four erect humeral setae present on promesonotum, otherwise promesonotum dorsally with regular subdecumbent pubescence; petiole and postpetiole with a pair of short erect setae posterolaterally, and shorter suberect, decumbent or appressed pubescence; abdominal tergites four to seven with very sparse short erect pilosity that is more abundant on sternites four to seven, and dense appressed to decumbent pubescence throughout. Color yellowish-brown to dark brown.

#### Queen

*unknown*.

#### Male measurements (n = 1)

HW 0.67, HL 0.46, EL 0.28, SL 0.11, MSNW 0.76, MSNL 0.72, WL 1.30, SPL 0.00, PTH 0.17, PTL 0.20, PTW 0.23, PPL 0.15, PPW 0.27, LHT 0.64, CI 1.46, OI 0.62, SI 0.24, MSNI 1.06, SPI 0.00, PTHI 0.82, PTWI 1.14, PPI 1.80, LBI 2.02.

#### Male description (Fig. 31A−C)

Small (HW 0.67, WL 1.30). Masticatory margin of mandibles with three teeth; clypeus only moderately protruding; eyes large (OI 0.62) and protruding, midline of eyes situated well below midline of head, almost extending to clypeal margin; antennae 12-segmented, scapes very short (SI 0.24); head much wider than long (CI 1.46); in full face view ocellar triangle situated at posterior head margin and elevated with respect to rest of face; occipital carinae distinct.

Mesosoma compact (MSNI 1.06, WL 1.30); mesoscutum in dorsal view as wide as long, posterolaterally constricted just before axillae; axillae tranversely compressed and narrow; scutellum in dorsal view broadly tapering from anterior to posterior end, dorsoposterior portion rounded; metanotum not distinctly protruding from below scutellum; dorsal face of propodeum very short, posterior face longitudinally convex; propodeal spines absent; petiole in dorsal view oval and laterally rounded, in lateral view petiole tapers greatly anteriorly; subpetiolar process absent; postpetiole lacking median impression; wings with a brownish hue.

Head sculpture shiny, rugulose within ocellar triangle; clypeus longitudinally with one short median and a pair of lateral carinae, and two short transverse carinae on each side; mesoscutum and scutellum shiny, propodeum longitudinally carinulate; petiole and postpetiole rugulose-shiny; scattered short erect pilosity on face; mesoscutum with scattered short erect pilosity; scutellum dorsolaterally with longer erect pilosity; petiole and postpetiole with longer dorsoposterior setae, and abundant erect pilosity dorsally and laterally; abdominal tergites and sternites four to seven with abundant short suberect pubescence, longer erect pilosity lacking. Color brown.

#### Variation

Workers of this species vary greatly in size, with the smallest specimens being found at the southernmost edge of the distribution (P.N. Bemaraha and Beanka).

#### Distribution and biology

*Crematogaster tsisitsilo* is found mainly in dry forest habitats in north-western Madagascar (Fig. 31D). Disjunct from this distribution, the species has also been found in the Forêt d’Antsahabe, a dry forest in the far northern tip of Madagascar. Sympatry with the *C. kelleri*-group exists only with *C. kelleri*. Not much can be said about the natural history of this species, since most collections were composed of foraging workers. One collection has been made from a carton nest, but the generality of this habit needs to be confirmed with additional data.

#### Etymology

This species is named for its complete lack of propodeal spines, as “tsisitsilo” means “no spines” in Malagasy. This name is to be treated as noun in apposition.

## Discussion

In this study, we were able to resolve yet another taxonomically difficult group of ants in Madagascar, the *Crematogaster kelleri*-group. We have demonstrated high levels of continuous morphological variation within *C. kelleri* and concluded that the former does not correlate with observed genetic variation or geography. Similar levels of variation have been described for other widespread taxa of *Crematogaster* in Madagascar, namely the *C. hova*-complex [11] and *C. ranavalonae* [12]. Nor is the bivariate coloration in *C. kelleri* (and *C. hazolava*) an isolated case. Within the *C. hova*-group, the dry forest species *C. grevei* has two color forms, one orange and one brown or black, the former being prevalent in dry deciduous forest and the latter in the spiny forests of south-western Madagascar [11]. Presumably habitat-specific selection processes favor one color form over the other. Particularly interesting in the case of *C. kelleri* is that these color forms co-occur at several localities. Whether these color forms still interbreed freely or may be on the way to reproductive isolation is an issue that remains to be investigated in a population genetic context.

The molecular sequence divergence in COI for *C. kelleri* is fairly high, ranging up to 14%, but this number is similar to levels in other *Crematogaster* species. More surprising and unusual is the low to almost absent sequence divergence in the ‘yellow clade’ (Figs 1 and 4). Most taxa within this clade stem from geographically close localities on the upper east coast of Madagascar. But because a few specimens also originate from a cluster of sites ca. 400 km further south, this low degree of divergence cannot be explained by geography alone. Although we considered the possibility of pseudogene (NUMTs [40], [41]) sequences being responsible for this pattern, we found no evidence in the relevant sequences, such as heteroplasmy or stop codons. A possible explanation for low divergence could be the presence of a “supercolony” originating from a bottleneck population [42], [43]. Supercolonies are prevalent in invasive, non-native ants [44], [45], but a re-introduction to a habitat by a population of a native species could leave a similar molecular footprint.

Distributions of the much less common remaining species within the *C. kelleri*-group are restricted to humid forests, and once more emphasize the elevated species richness (but not necessarily abundance) of this genus in Madagascar’s eastern rainforests, especially in the northern tip of the island. The newly described *Crematogaster tsisitsilo* presents an interesting exception to this common pattern, as it is found in dry forests in the north-western region, as well as at a disjunct locality in the far north. The isolated phylogenetic position of this taxon in Madagascar [9] suggests that this species either is a recent addition to the Malagasy ant fauna or its closest relatives in Madagascar have gone extinct. The notion that this species is not endemic to Madagascar and already has been described from mainland Africa needs to be considered. Although we have reasonable confidence that this is not the case, the taxonomic state of African *Crematogaster* is in such chaos that we cannot fully exclude this possibility.

This study underlines once more the importance of utilizing molecular methods, as well as geographical data, to better understand morphological variation in ants. In conclusion, we hope our approach inspires more such revisions employing multiple types of data analysis, and supports further research on Madagascar’s extraordinary insect and ant fauna.

## Supporting Information

Table S1 **Full collection data of molecular voucher specimens.** Voucher specimens, Genbank accession numbers and locality data of all specimens included in the molecular study.
(XLSX)

Table S2 **List of material examined.** Full list of locality data for all specimens examined within the morphological study.
(XLSX)
